# The Influence of Topographic and Dynamic Cyclic Variables on the Distribution of Small Cetaceans in a Shallow Coastal System

**DOI:** 10.1371/journal.pone.0086331

**Published:** 2014-01-22

**Authors:** Marijke N. de Boer, Mark P. Simmonds, Peter J. H. Reijnders, Geert Aarts

**Affiliations:** 1 Department of Ecosystems, Institute for Marine Resources and Ecosystem Studies, Wageningen UR, Den Burg, The Netherlands; 2 Department of Aquatic Ecology and Waterquality Management, Wageningen University, Wageningen, The Netherlands; 3 Science Department, Whale and Dolphin Conservation, Chippenham, Wiltshire, United Kingdom; University of Waikato (National Institute of Water and Atmospheric Research), New Zealand

## Abstract

The influence of topographic and temporal variables on cetacean distribution at a fine-scale is still poorly understood. To study the spatial and temporal distribution of harbour porpoise *Phocoena phocoena* and the poorly known Risso’s dolphin *Grampus griseus* we carried out land-based observations from Bardsey Island (Wales, UK) in summer (2001–2007). Using Kernel analysis and Generalized Additive Models it was shown that porpoises and Risso’s appeared to be linked to topographic and dynamic cyclic variables with both species using different core areas (dolphins to the West and porpoises to the East off Bardsey). Depth, slope and aspect and a low variation in current speed (for Risso’s) were important in explaining the patchy distributions for both species. The prime temporal conditions in these shallow coastal systems were related to the tidal cycle (Low Water Slack and the flood phase), lunar cycle (a few days following the neap tidal phase), diel cycle (afternoons) and seasonal cycle (peaking in August) but differed between species on a temporary but predictable basis. The measure of tidal stratification was shown to be important. Coastal waters generally show a stronger stratification particularly during neap tides upon which the phytoplankton biomass at the surface rises reaching its maximum about 2–3 days after neap tide. It appeared that porpoises occurred in those areas where stratification is maximised and Risso’s preferred more mixed waters. This fine-scale study provided a temporal insight into spatial distribution of two species that single studies conducted over broader scales (tens or hundreds of kilometers) do not achieve. Understanding which topographic and cyclic variables drive the patchy distribution of porpoises and Risso’s in a Headland/Island system may form the initial basis for identifying potentially critical habitats for these species.

## Introduction

Due to the elusive nature of most small cetacean species, understanding their habitat selection can be challenging. This has led to the development of more indirect methods, where the heterogeneity in distribution is quantified as a function of habitat variables, such as water depth, sea surface temperature, primary productivity, bottom type, tidal currents and frontal systems [1]–[4]. Such habitat models play a key role in improving the understanding of the ecological processes underlying cetacean distributions [5], [6].

Most cetaceans tend to be wide-ranging and their abundance is typically studied using large-scale line-transect surveys that provide a single large-scale ‘snapshot’ of the distribution [7]. Such studies are not designed to study the fine-scale heterogeneity in high-density areas and they do not provide detailed information regarding temporal drivers that might influence the distribution of cetaceans. Studies that focus on the habitat selection of a cetacean species therefore do this at a much smaller scale (0.5×0.5–4×4 km^2^) using either a dedicated research vessel or Platform of Opportunity [1], [2], [8], [9].

Several cetacean species are often encountered close to islands and headlands where temporal drivers, such as strong tidal currents can play a dominant role [9]. Such locations may provide an excellent opportunity to install low-cost observation platforms to carry out dedicated (effort-corrected) surveys. An appealing aspect of such land-based surveys is that they can capture the variations in occurrence of cetaceans in both space and time at a reduced cost compared to boat-based studies. The objective of this study is to provide a temporal insight into the fine-scale spatial distribution that studies conducted over broader geographic scales do not achieve. We focus here on the harbour porpoise *Phocoena phocoena* and Risso’s dolphin *Grampus griseus*, which both occur in Welsh waters, and are regularly sighted from Bardsey Island in North Wales (United Kingdom) [10]. Opportunistic records of Risso’s dolphins made from Bardsey Island (1976–2005) indicate that this species primarily occurs here during the months of July to October with additional sightings recorded in April (de Boer, unpublished data). The harbour porpoise is sighted here year around and only occasional sightings are made of other cetacean species [10], [11]. The cetacean community off Bardsey Island is therefore best described as dominated by porpoises and Risso’s dolphins.

Like any other headland/island system, Bardsey Island acts as a flow obstacle which leads to the formation of residual eddies on either side of the island during flood and ebb [12], [13]. At fine spatial scales, small-scale eddies and fronts appear to enhance the primary productivity and it is recognised that such features may concentrate prey [14], [15]. Prey aggregations within headland and island wakes are believed to result from complex secondary flows which concentrate plankton near the surface at convergences and at the edges of eddies [16]. There have been few studies of cetaceans foraging in island/headland wakes. Johnston et al. [9], [17] reported on fin whales *Balaenoptera physalus,* minke whales *Balaenoptera acutorostrata* and harbour porpoises that exploited a tidally driven island system in the Bay of Fundy. In the Moray Firth (Scotland), bottlenose dolphins *Tursiops truncatus* showed fine-scale foraging movements within a narrow channel [18]. In Alaska the abundance of humpback whales *Megaptera novaeangliae* appeared to be related to tidal influences near headland wake systems [19]. Pierpoint [20] and Isojunno et al. [21] reported on porpoises in a headland/island system in South Wales.

The area that includes Bardsey Island and its surrounding waters is located in the northern part of Cardigan Bay and has been designated as a Special Area of Conservation (SAC), meeting the requirements of the EU Habitats and Species Directive [22]. This regional SAC, also called ‘Pen Llŷn a’r Sarnau’ was designated for a number of features including estuaries, coastal lagoons and reefs and also the grey seal *Halichoerus grypus* and bottlenose dolphin. Risso’s dolphins are listed under Annex IV of the EU Habitats and Species Directive. Annex IV species, which include all cetaceans, are afforded ‘strict protection’ whereby the deliberate capture, killing and disturbance of these species are strictly prohibited (Council Directive 92/43/EEC). Harbour porpoise and bottlenose dolphin are the only two species of cetaceans listed under Annex II which are afforded the designation of SACs whereby ‘the viability, population size and range of a species’ should be maintained in the long term (Council Directive 92/43/EEC). However, no SACs have been designated for harbour porpoise in the UK, although sites have been designated in other parts of Europe. A better understanding of how the distributions of small cetacean species are changing in space and time, at different scales, will ultimately aid the selection of protected areas.

In this study, we investigated whether localised areas afford temporary but predictable habitat for harbour porpoises and Risso’s dolphins. We use long-term data from fixed viewing points located on Bardsey Island. By constructing habitat selection models we explore whether these localised areas (or hotspots) are influenced by dynamic cyclic variables (e.g. tidal and lunar cycles) and topographic variables. As such, the study provides a temporal insight into the fine-scale spatial distribution of two species beyond the resolution of most studies and management considerations.

## Materials and Methods

### Survey Area

**Figure 1:**
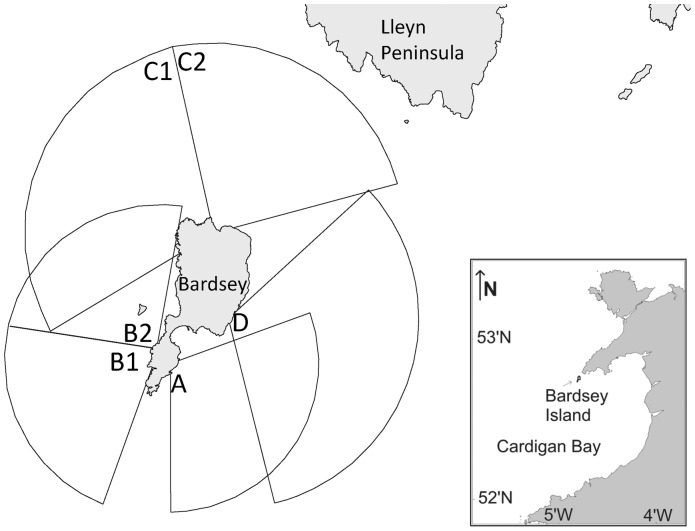
The location of Bardsey Island within Cardigan Bay (Wales). The four different viewing points (A-D) and corresponding survey sectors are also shown.

**Figure 2:**
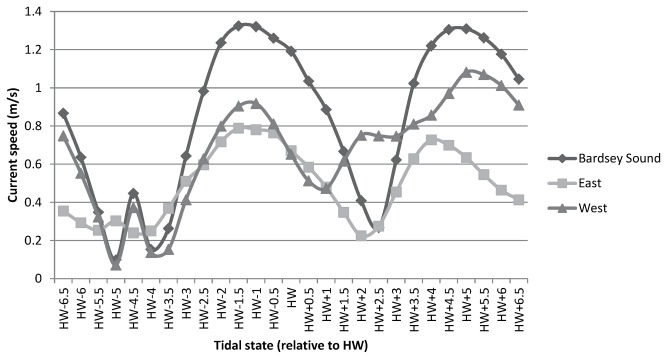
The dominating current speed (m/s) measured for Bardsey Island. The current speeds for different areas to the West, East and to the North (Bardsey Sound) of Bardsey Island are shown.

Cardigan Bay is a large shallow embayment on the East side of the St. George’s Channel entrance to the semi-enclosed Irish Sea Basin. Within the Cardigan Bay, lies the Lleyn peninsula (Wales), which is orientated Northeast/Southwest and is some 40 km in length, ending in a headland adjacent to deeper water. Bardsey Island (with dimensions of 2.6 km by 1 km) is situated off the tip of the Lleyn Peninsula in the northern part of Cardigan Bay at 52°45.36′N and 004°47.17′W and is separated by Bardsey Sound (approximately 3 km wide; Figure 1). Bardsey Island is owned and managed by the Bardsey Island Trust. The tides along the coast of the Lleyn Peninsula are extremely rectilinear and mainly semi-diurnal in character [12]. There are strong tidal currents that exist in the waters surrounding Bardsey Island which have currents of up to 3 m s^−1^ (6 knots) [12]. Water is driven through Bardsey Sound by the tidal current as it enters and exits the Irish Sea during the semi-diurnal tidal regime. The tidal flow through the survey area is mainly Northwest (i.e. flowing through the Sound) during flood and is Southeast for the remainder of the tidal cycle. Interestingly, during HW the mean current speed is still at its highest. Low Water Slack tide (LWS) occurs on the West and North side of the Island between HW−5.0 and HW−3.5 hrs. The High Water Slack tide (HWS) to the West occurs between HW+0.5 and HW+1.5 hrs but to the North this occurs later (between HW+2 and HW+3). To the East of the Island, LWS and HWS occur between HW−5 to HW-4 hrs and between HW+1.5 to HW+2.5 hrs respectively (Figure 2). Bardsey Island constitutes an obstacle to these tidal streams and an island ‘wake’ is formed behind it, causing eddies and overfalls, especially on high tides. The race on the flood tide sets rapidly after LWS to the West (Figure 2). According to Pingree and Griffiths [23] the waters to the West are mixed and to the East are transitional, with a frontal system that exists in the shallow Cardigan Bay area in summer which is highly susceptible to wind mixing.

### Land-based Survey Design

A standardised method (‘scan sampling’) was used that was sensitive to short-term changes in the number of cetaceans. No permits were required for the described study, which complied with all relevant regulations. Observations were carried out during the summer months between 2001 and 2006. A study area (sighting angle up to 90°–115°) was slowly scanned using 7×50 Nikon binoculars for a period of 10 minutes [20]. Whenever possible, simultaneous observations were carried out from four observation points which varied in height and survey area (Figure 1). We produced a series of 10-minute ‘snapshots’ for each sampling segment, detailing the location of cetaceans sighted. To account for tidal amplitude (±5 m at spring tide), the height of the observation point above sea level was calculated using tidal height corrected for Bardsey Island (WXTide32 version 4.7). Points A and B (both at 17 m height at LW) were situated at the southern tip of the Island. Point B overlooked waters with exposure to prevailing south-westerly wind and wave action and containing complex bathymetric features, whereas point A overlooked a leeward habitat. The higher points (C–D) were situated at heights of 38 m and 60 m at LW respectively and were located on the northern part of the Island. Point C covered two survey areas of which one overlooked the waters in Bardsey Sound with strong tidal streams and the other area overlooked the western part of the Island which partly overlapped with an area covered from point B. Point D overlooked the eastern part of the Sound but also partly overlapped with the leeward habitat covered from point A. Because points B and C were wide-viewing points, two different survey sectors were covered, totalling the number of similar-sized survey sectors surrounding the Island to six (Figure 1).

Observers switched scanning every 10 minutes and also changed platforms every 2–4 hours to prevent observer exhaustion and to address any observer bias. The following information was collected with each sighting: radial distance (using reticule binoculars), bearing (using the built-in compass in the binoculars – these were frequently checked and calibrated), surfacing direction, group-size, presence of calves and juveniles. Surfacing speed was described as either: ‘slow’–a lethargic-type roll; ‘moderate’–a typical porpoise surface roll with back and upper flanks visible; or ‘fast’–exposing much of the head and flanks and creating some spray. Distinctive behaviours were noted separately. For each 10-minute scan various environmental details were noted, including the Beaufort sea state (0–4) and visibility (poor, moderate, good, excellent). Optolyth telescopes (x30) were used to aid group-size estimation and to distinguish juveniles and calves.

### Data Analysis

We estimated the position of each sighting using the location of the viewing platform (X and Y coordinates), the bearing, radial distance (using the GEOFUNC Software with spherical trigonometry functions) [24] and observation height (taken into account the tidal amplitude according to WXTide32 version 4.7; set-location Bardsey Island). These were imported into ArcGIS version 10 with the following coordinate system (from now on referred to as Bardsey Projection): Transverse_Mercator; Central_Meridian: −4.785; Latitude of Origin: 52.75543; Linear Unit: Meter; Geographic Coordinate System: GCS_WGS_1984.

### Detectability & Precision of Measurements

It is extremely unlikely to expect that all animals within a surveyed area to be sighted and both habitat preferences and distance can influence the detection function. The ability of the observer to sight the animal is negatively affected by increasing distance between the animal and the observer. When studying the habitat preferences of cetacean species, it is assumed that spatial variations in sighting rate are the result of differences in habitat use rather than any potentially confounding variables, such as the distance from the observer. Estimating the distance related detection probability would be possible by using data collected from two-independent land-based observers [25] however such data were not collected in the present study. Instead, to control for this effect, we took a more conservative estimate and removed all locations outside a given radius around each of the observer platforms which defined the point at which distance from the observer starts to influence the likelihood of detection rather than habitat preferences. In order to estimate this radius, we studied the effect of distance on the detectability of sightings by plotting an accumulation curve which shows the proportion of total number of sightings up to a given distance (Supplementary Figure S1). This allowed us to estimate the ‘inflection point’, which is the point marking the distance at which there is a change from constant to declining detection rate with distance (the point where the increase changes from linear increase to a curvilinear increase). Since small cetaceans are notoriously difficult to observe with high sea states, a similar comparison was made in order to determine which sea states followed a similar accumulation curve (for each survey site) and could be pooled for analysis (i.e. which of the higher sea states needed to be excluded to reduce bias in the ability of detection; Supplementary Figure S2).

We assessed the precision of measurements by looking at the level of error from rounding to the closest 0.5 reticles on the binoculars.

### Identifying Areas of High Density

The kernel estimated probability of an animal using the habitat at a specified location is a smoothed function of all sighting locations within a specified range (neighbourhood/bandwidth) around that location [26], [27]. This method is therefore less affected by errors on the exact locations of an animal’s position than some other space-use estimators [28]. The kernel density estimator is extremely sensitive to the choice of smoothing parameter (bandwidth) and it is recommended that a smoothing bandwidth that is at least equal to the uncertainty in the location is used [27].

To identify key habitats for harbour porpoises and Risso’s dolphins, kernel density estimation grids were produced in ArcGis V10 using the fixed kernel density estimator (‘kde’ commands) by means of Geospatial Modelling Environment (SpatialEcology.com). The Gaussian (bivariate normal) kernel was used where the optimized bandwidth matrix was estimated via smoothed cross validation (SCV) and set to an output cell size of 50×50 m. This was found to best relate to the resolution of the habitat variables and our fine-scale analysis. The selected value of 50 m also was appropriate considering the error on the majority of sighting position estimates.

The relative size and form of the kernel density estimate is dependent on the total number of locations and their distribution. More survey effort and increased sightability generally leads to more sightings. Therefore, we treated the data from each observation point separately (due to differences in height and effort), and, to compensate for differences in the amount of survey effort in each of the survey areas we randomly selected those periods when effort was conducted from all four observation points resulting in the same number of scans for all survey sectors (*n* = 600). Each sighting falling in overlapping areas received a weight of 0.5 to adjust for double-effort in these areas. We then carried out kernel analysis for each species in order to identify the areas of highest density of sightings for each sampling area. In those areas that overlapped we expected to identify the same areas of high density which helped confirming the findings from any one site.

By determining the smallest possible area containing user-specified percentages of the locations, the kernel grid was divided into percentage volume contours for 95%, 75%, 60% and 50% intervals. This means that the area within the 50% contour represents areas with highest density and the 95% contour almost the entire range. The kernel density estimation tool does not give the possibility of excluding land.

### Environmental Variables Used to Study the Habitat Preferences

Acoustic Doppler Current Profiler data (ADCP) were obtained from the University of Bangor at a 300 m×300 m resolution over complete tidal cycles [12]. This data included tidal current measurements in Bardsey Sound and around Bardsey Island and were collected during a survey using a ship-borne ADCP combined with direct reading and moored current meters [12]. The ADCP observations were normalized by the tidal range and then scaled to high spring conditions for the nearest Port Liverpool. From the ADCP data, maps of surface currents at the different tidal states in respect to HW at Liverpool were derived from 6 hours prior to 6 hour after HW, at 30 minute intervals (see [12] and references therein).

Tidal current data were manually interpolated with respect to HW at Bardsey Island as follows. Every ten minutes the tidal state (hours after high water) and tidal height (meters above extreme low tide) was obtained from the tidal prediction programme (WXTide32 version 4.7; set-location Bardsey Island).

A range of environmental variables were available for inclusion in the analysis including temporal/tidal variables and topographic variables: *The X and Y coordinates* (Bardsey grid projection) were included using the estimated sighting positions. *Depth values* were obtained as an ASCII grid (50 m×50 m resolution) from the offshore digital dataset (United Kingdom Hydrographic Office/Marine DigiMap; ©Crown Copyright/SeaZone Solutions Ltd 2008). From these grids, *distance to coast*, *seabed slope* (0 to 90°), standard deviation *(SD) of slope* (used as a measure of spatial variation in bottom topography and this was calculated for each cell and the 5 surrounding cells in Arcview GIS 10.0) and *aspect* (i.e. the azimuthal direction in which a tangent plane faces, 0 to 360°) were calculated using Spatial Analysis tool functions in ArcGIS (version 10). Temporal variables such as *day of year, hour of day* and *year* were included. Temporally varying tidal variables were also included, such as *tidal state* (the time in the tidal cycle relative to High Water) and *lunar cycle* (the number of days before (i.e. negative values) or after (i.e. positive values) neap tide, i.e. the date of the lowest change in tidal height) and spatially varying tidal variables were also used, such as *tidal current speeds* and *current directions* and *tidal stratification*. These tidal variables were calculated for each tidal state (i.e. from 6 hours prior to 6 hour after HW, at 30 minute increments). In order to reduce the number of covariates, we did not account for the fact that there are two neap tides and two spring tides within a single month and that these are different in terms of tidal ranges.

*Spatial variation of current speed* was estimated as follows: Based on sines and cosines rules, current speed in North-South (Y) and East-West (X) direction was calculated using the available data on current speed (m/s) and direction (degrees). The spatial variation in each of the two current directions (i.e. *SD_X_* and *SD_Y_*) was calculated for each grid cell by estimating the standard deviation of that cell and the 5 surrounding cells. Finally, the *average spatial variation in speed* was estimated by applying the Pythagorean equation on *SD_X_* and *SD_Y_*.

In shallow seas (<200 m) the tendency of a water column to thermally stratify can be quantified by the ratio between the total depth (*h*) and the cube of a measure of the tidal current amplitude (*U*), *h*/*U* ^3^ [23], [29]. Tidal stratification, *log10(h/U^3^),* was found to be the best indicator of the probability of presence and abundance of individual marine apex predators (including harbour porpoise) [30]. *Tidal stratification* values were calculated over the whole study area using the depth data (resolution 50 m, see above) and the tidal velocities from the ADCP data (resolution 300 m). The *mean stratification* was also computed using the mean tidal velocities calculated from the ADCP data over one complete tidal cycle [30].

### Statistical Modelling

We assumed that all sightings up to the inflection points were detected. We used sea states 0–2 for habitat modelling regarding dolphins and sea state 0–1 for porpoises and only included those porpoise observations made during sea state 2 up to the corresponding inflection points (Supplementary material S1). Re-sightings of animals within one 10 min scan were omitted. Some individuals observed in one scan could be re-sighted in the following scan, however since individuals can move several hundreds of meters between two scans, it was not possible to reliably identify re-sightings as such. We therefore regarded each scan as a new independent sampling unit. However possible correlation between scans within a day was taken care of in the model selection procedure.

The distribution of harbour porpoises and Risso’s dolphins was modelled as a spatiotemporal Inhomogeneous Poisson Point process (IPP) [31], [32]. Under an IPP, the individual animals are treated as point observations in space and time. To quantify variations in density, these observations were contrasted with where and when animals could have been observed, taking into account the variations in effort. This was achieved by sampling uniform random within the survey area up to the distance of the inflection point (to reflect uniform detection probability and following the same assumptions as for the presence points) at times when survey effort took place at the observation platform in question. For each 10 min scan we created two ‘absent’ or availability points, corresponding to approximately 16 thousand points, which were used for approximating the IPP process likelihood function. Next, an infinitely weighted logistic regression (IWLR) [33] was fitted to the data. Here, the animal observations were treated as response of 1, and the contrasting availability points were treated as a response of 0. The variations in the response were modelled as a function of environmental variables. All potential explanatory variables were screened using histograms, dot plots (univariate) and scatter plots (bivariate) to determine distributions, detect outliers and identify co-linearity between variables. Where 2 variables were strongly collinear (r ≥0.8), one was excluded from further analysis [30]. Initial exploration of co-linearity between the proposed model covariates found high correlation (r ≥0.8) between distance-from-coast and mean-stratification, radial-distance and mean-stratification, and also between tidal-stratification and current-speed. The predictor variables distance-from-coast, radial-distance and current speed were removed (as tidal-stratification and mean-stratification were considered to be the more biologically relevant variables) [30], [34].

The potential environmental covariates used in the model were a thin plate smooth [35] of mean- stratification, tidal stratification, day of year, hour of day, year, depth, slope, spatial variation in slope and spatial variation in current speed. The variables lunar cycle, tidal state, aspect and current direction are circular covariates, and therefore were included as cyclic cubic regression splines (type “cc” in the R-package mgcv). At the data extremes the estimated smooth function is identical up to the 2^nd^ order derivate [35]. Therefore the data points located around both extremes (e.g. for aspect 0 and 360 degrees) contribute to the estimation of the smooth function on either side. Here, we made the implicit assumption that each point in space is a unique habitat and we therefore included a tensor product smooth of X and Y coordinates (Bardsey Projection) in the model. Although, X and Y cannot have a direct causal relationship with the underlying process of habitat selection, they may correlate spatially with environmental variables that do. This tensor product smooth can therefore absorb large-scale residual spatial effects in the distribution of sightings that cannot be explained by the environmental variables included in the model. Furthermore, the inclusion of X and Y will also deal with any issues regarding unbalanced sampling effort although the IPP process also accounts for any differences in effort between the various observation points. Finally, sea state and viewing point were included as a factor variable because it was expected that these would affect the distribution of sightings.

Forward model selection was carried out using likelihood-based k-fold cross-validation [36], [37]. All animal and control observations were grouped by day, and a model was fitted using all data, except for one day (i.e. the left-out data). Next the resulting model was used to predict for the left-out day and to estimate the corresponding likelihood. This was repeated for all *k* days and all variables. The model with the lowest overall cross-validation likelihood was retained for further analysis.

## Results

### Detectability & Precision of Measurements

We studied the effect of distance on the number of Risso’s dolphin and harbour porpoise sightings by plotting accumulation curves which showed the proportion of total number of sightings within a given distance (Text S1 and Text S2). It was also found that the accumulation curves for either Risso’s dolphins or porpoises differed for observation platform C (C1 *vs* C2) and it was decided to treat these two survey sectors separately because of their different inflection points (Supplementary Figure S1). The accumulation curves for both sectors (B1 and B2) covered from observation platform B were comparable and we concluded that data could be pooled. We then explored how the sea state was affecting the accumulation curve for both species (Supplementary Figure S2). On the basis of the outcome of these investigations, we were able determine the distance (based on the defined inflection points) to which we assume that the number of sightings remained constant at each different sea state (Supplementary Table S1) for each of the different survey sectors (A, B, C-1, C-2 & D) and for both species.

The step-wise appearance of the accumulation curves and concentric circles in the distribution of Risso’s (and to a lesser extend porpoise) sightings is most likely caused by the inaccuracy of the inclination and the angle measurements made using the reticule binoculars and the built-in compass (where rounding occurred to the nearest half reticule and the nearest degree). This step-wise appearance is to some extend reduced when accounting for the tidal amplitude which affects the observation height of platform and thereby the estimated radial distances to sightings. The distance measurements per 0.5 reticules are shown in Supplementary Figure S3. It is evident that for larger distances the difference between two subsequent 0.5 reticule steps is large, however, within 1.5 km the difference is <100 m and within 1 km this is <50 m (Supplementary Figure S3). Overall, the error was small for porpoises as the fast majority of sightings occurred at distances <1500 m (91% of all sightings) or <1100 m (70.3%). On average the location error was higher for dolphins, because the dolphins were typically sighted at greater distances (with 58% of dolphin sightings occurring at a distance >1500 m).

### Effort & Sighting Rates

We used sea states 0–2 for any data analysis regarding dolphins and sea state 0–1 for porpoises and only included those porpoise observations made during sea state 2 up to the corresponding inflection points (Supplementary material S1). After filtering for sea state and taking into account the different inflection points, a total of 791 porpoise and 238 Risso’s dolphin sightings were included in the data analysis with a total effort of 8262 scans of 10 minutes each (Table 1). Most effort was conducted from point D, not only because this site offered the most sheltered study area but also because this was the only look-out manned during those periods when only two people were stationed on the island. As such, this observation point collected the most data during July, whilst the other observation points had the majority of effort carried out in August and September (Table 1).

**Table 1:** Overview of different survey sites regarding height, sector coverage (size) and summary of systematic effort (number of 10-min scans) during sea states 0–2 with number of harbour porpoise and Risso’s dolphin sightings relative to corresponding inflection points.

Surveysite	Site specificsHeight(Size)	Effort(scans)	Harbour porpoiseSightings(animals)	Risso’s dolphinSightings(animals)	EffortJuly(Scans)	EffortAugust(Scans)	EffortSeptember(Scans)
A	17 m (110°)	900	62 (104)	0	155	531	214
B	17 m (2×90°)	887	16 (28)	33 (57)	107	475	305
C-1	37.5 m (110°)	805	28 (63)	174 (242)	124	300	381
C-2	37.5 m (90°)	1486	180 (371	22 (68)	262	601	623
D	60 m (115°)	4183	505 (856)	9 (33)	1227	2037	919
TOTAL		8261	791 (1422)	238 (400)	1875	3944	2442

### Tidal Cycle

**Figure 3:**
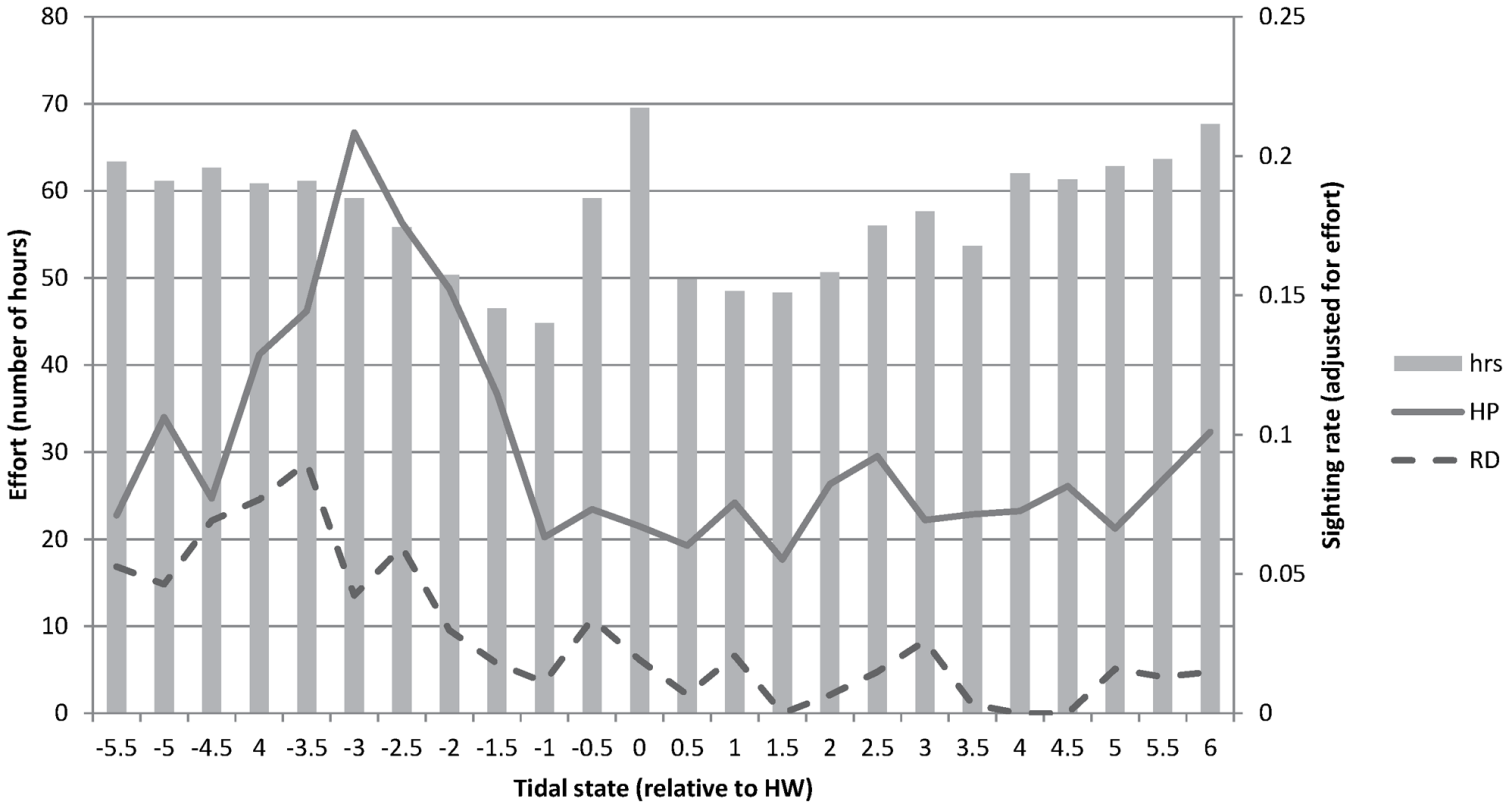
Hours of effort and sighting rates for different tidal states. HP = Harbour porpoise; RD = Risso’s dolphin.

For each tidal state more than 45 hrs of effort was carried out. The sighting rates for dolphins and porpoises (adjusted for effort) showed a peak at HW-3.5 and HW-3 respectively (Figure 3). This is approximately one hour after Low Water Slack when the currents change direction from SE to NW. A smaller peak in sighting rate for porpoise is evident during the next slack water period (HWS: HW+1.5 until HW+2.5).

### Identifying Areas of High Density

**Figure 4:**
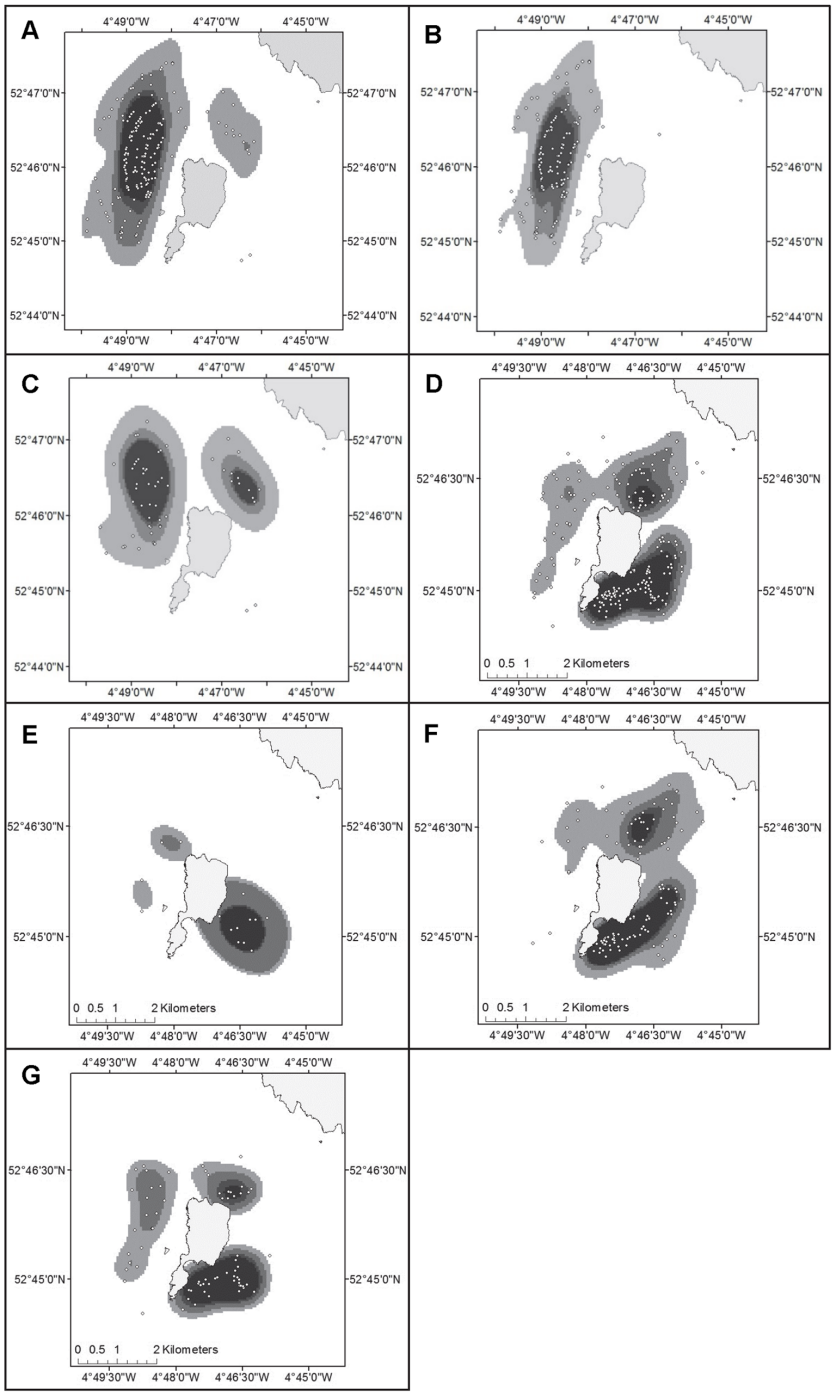
Kernel density utilisation grids. Risso’s dolphin: All data (A); August (B); September (C) and Harbour porpoise: All data (D); July (E); August (F); September (G). Densities are presented in percentiles (50; 60; 75; 95%). Sighting locations are indicated by small circles.

Kernel methods were used to analyse spatial clustering in the sightings data and the resulting 50% density isopleth was selected to define the core areas. From the kernel density percent volume contours it is evident that the survey area is not evenly utilised by both species (Figure 4). The Risso’s dolphins use a core habitat to the West of the island, and this area is used both in August and September (Figure 4). An area to the North of the Island (within Bardsey Sound) is also used in September (Figure 4C). Harbour porpoises use a different area to that of Risso’s dolphins, although there is a noticeable overlap where both species occur within the sound in September. The area to the East of the Island, and also an area within the Sound, are identified as core areas where porpoises regularly occur (Figure 4D). In August, the majority of porpoises occur to the East of the Island (Figure 4F). The area within the Sound is more pronounced in September but is located slightly closer to the shore (Figure 4G). In addition, there is more porpoise activity in September to the West of the Island overlapping with the area where Risso’s mainly occur. The 50% kernel volume contour for porpoises to the East of the Island involved an area of 2.8 km^2^ and in the Sound this was 0.9 km^2^. For Risso’s dolphins the core area involved an area of 2.6 km^2^. These represent 19%, 6% and 8% respectively of the full survey area of 34.31 km^2^ (for dolphins) and 14.6 km^2^ (for porpoises).

Using the kernel density plots we checked to see if the occurrence of harbour porpoise and Risso’s were correlated and found no evidence for this (*R^2^ = −0.2309*), suggesting that the two species use the local spatial area in different ways. A Mann-Whitney U test also confirmed that the kernel density data were significantly different between the two species (p<0.0001).

### Habitat Modelling

The final habitat model for harbour porpoises, selected through forward stepwise model selection, contained sea state, a spatial smooth of X- and Y-coordinates, the observation site, lunar cycle, mean stratification, day of year, depth, aspect, tidal state and slope (Table 2). The model explained only 7.5% of the deviance in the observed variation in the response variable (Supplementary Table S2). Sea state was the most important variable and was retained first (Supplementary Table S2). The parameter estimates for sea state 1 and 2 (relative to sea state 0), were −0.58 and −1.69, respectively (see Supplementary Table S2). This implies that the sighting rate under these conditions was 0.56 (i.e. e^−0.58^) and 0.18 (i.e. e^−1.69^) lower, compared to sea state 0. The spatial smooth of X and Y coordinates was the second most important covariate to be retained, explaining more of the variation than any other spatial or temporal variable. The smooth of X and Y coordinates absorbs any residual large scale spatial pattern in marine mammal sightings that cannot be explained by the environmental variables included in the model. The apparent significance of this covariate (based on model selection), suggests that a biologically important variable that drives the porpoise distribution was not included this study.

**Table 2:** Forward variable selection based on models fitted to harbour porpoise data, based on the cross-validation log-Likelihood (CVLL).

Covariate	CVLL	ΔCVLL
Sea State	−9645.54	
te(X,Y)	−9524.84	120.70
Site	−9421.26	103.58
s(Lunar cycle)	−9336.61	84.65
s(Mean stratification)	−9305.75	30.86
s(Day of year)	−9285.25	20.50
s(Depth)	−9267.39	17.85
s(Aspect)	−9252.76	14.64
s(Tidal state)	−9241.04	11.72
**s(Slope)**	**−9240.30**	**0.74**
s(Year)	−9241.38	−1.08
s(Hour of day)	−9249.61	−8.23

ΔCVLL is the change in CVLL by adding a (smooth of the) covariate. Sea state and Site entered the model as factor variables. “te(X,Y)” represents a tensor product smooth of X and Y coordinates (Bardsey projection). “s” represents a thin plate regression spline smoother (or cubic regression spline for cyclic smoothers, i.e. for the covariates Lunar cycle, aspect and tidal state). The best model contained all variables up to slope.

**Figure 5:**
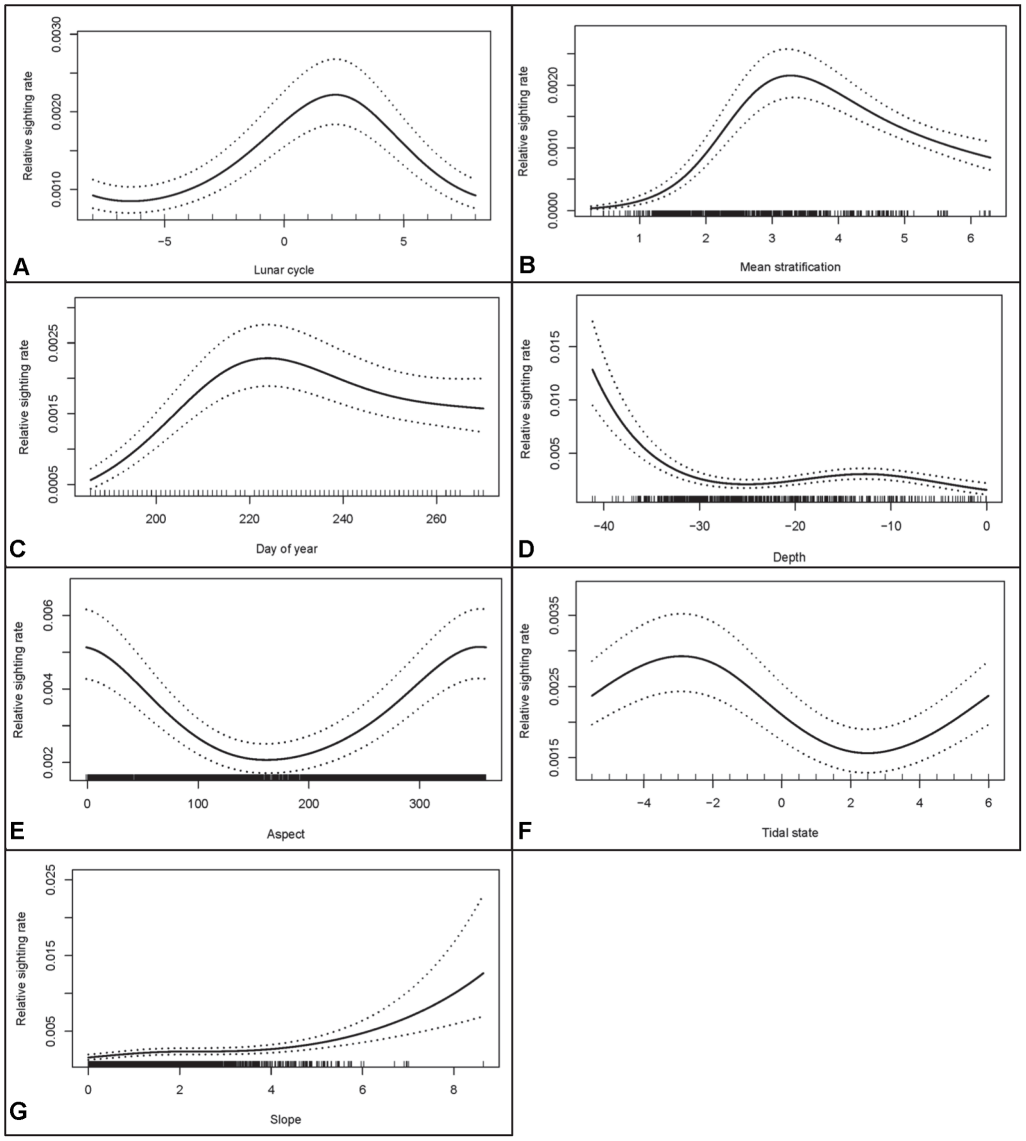
The estimated effect of environmental covariates on the observed harbour porpoise sighting rate. Predictions were made by varying the variable of interest (e.g. Lunar cycle in the first figure), but keeping the other values fixed at median values at which they occur in the model data.

**Figure 6:**
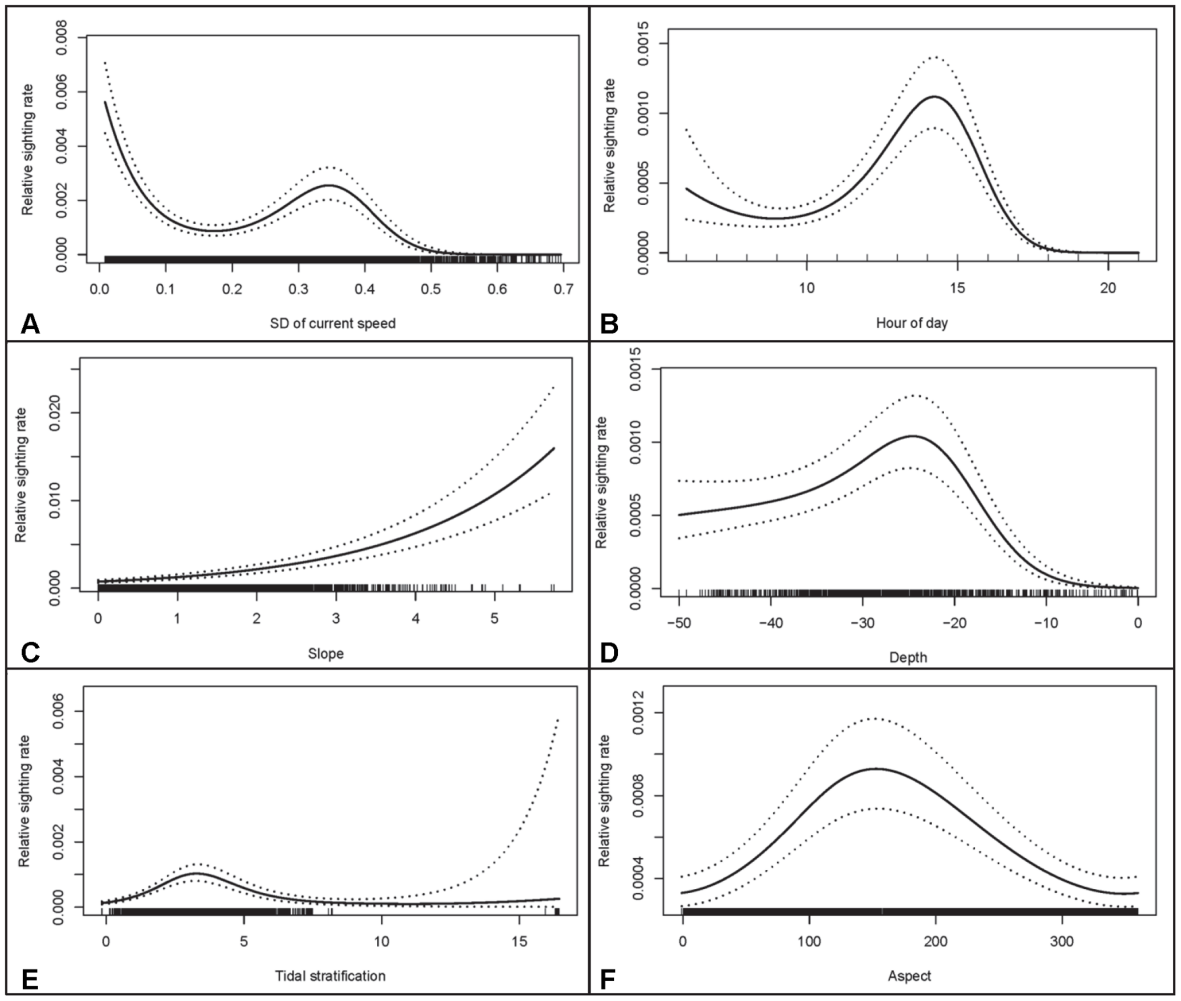
The estimated effect of environmental covariates on the observed Risso’s dolphins sighting rate. See Figure 5 for more details.

The final selected model also indicated that porpoises were more frequently seen 2–3 days following neap tide (Figure 5a), in areas with a relatively high stratification (3.3 m^−2^ s^3^; Figure 5b), and mostly in August (Figure 6c). Depth was the 7^th^ most influential variable, suggesting that the highest sighting rate occurred in areas of approximately ∼14 m depth or depths exceeding 30 m (Figure 5d). The perceived preference for increasing depths is mostly driven by a number of sightings in the deeper main channel. Finally, sighting rate was higher on NW facing slopes (Figure 5e), around approximately 3 hours before HW (Figure 5f) and steeper slopes (Figure 5g).

The final habitat model for Risso’s dolphins, selected through forward stepwise model selection, contained observation site, sea state, spatial variation of current speed, hour of day, slope, depth, tidal stratification and aspect (Table 3). The model explained 19.7% of the deviance in the observed variation in the response variable (Supplementary Table S3). Observation site was the most important variable and was retained first (Supplementary Table S3). The parameter estimates for points B, C1 and C2 relative to sites where no dolphin sightings were made, were 2.3, 3.6 and 2.2, respectively (Supplementary Table S3). This implies that the sighting rate from these observation sites was 9.99 (i.e. e^2.3^), 36.6 (i.e. e^3.6^) and 9.02 (i.e. e^2.2^) higher, compared to sites where no sightings were made (e.g. Point A). The sea state was the second most important covariate to be retained. The parameter estimates for sea state 1 and 2 (relative to sea state 0), were 0.65 and −0.67, respectively (Supplementary Table S3). This implies that the sighting rate for sea state 1 and 2 was 1.91 (i.e. e^0.65^) and 0.51 (i.e. e^−0.67^) times the sighting rate during sea state 0.

**Table 3:** Forward variable selection based on models fitted to Risso’s Dolphin data.

Covariate	CVLL	ΔCVLL
Site	−3036.86	
Sea State	−2953.44	83.42
s(SD of current speed)	−2906.52	46.92
s(Hour of day)	−2872.70	33.82
s(Slope)	−2855.48	17.22
s(Depth)	−2843.52	11.96
s(Tidal stratification)	−2834.70	8.83
**s(Aspect)**	**−2824.53**	**10.16**
s(SD of sloop)	−2840.46	−15.92
s(Current direction)	−2861.82	−21.36
s(Mean stratification)	−2892.66	−30.84
s(Tidal state)	−2927.68	−35.02

For more details, see Table 2. The best model contained all variables up to Aspect.

This selected model indicated that Risso’s dolphins were more frequently seen in areas with a low spatial variation of current speed (Figure 6a). The dolphins were most frequently seen in the afternoon (2pm; Figure 6b), in areas with a relatively steep slope (Figure 6c). Depth was the 4^th^ most influential variable, suggesting that the highest sighting rate occurred in areas of approximately ∼25 m depth (Figure 6e). The sighting rate for dolphins occurred in areas with a tidal stratification of ∼2.7 m^−2^ s^3^ (Figure 6f) and on south-facing slopes (Figure 6g). For illustrative purposes, Supplementary Figure S4 shows a visualisation of the predicted relative sighting rate for porpoises and dolphins and density plots for both the used and availability points are shown in Supplementary Figures S7 and S8.

## Discussion

This study showed that the Risso’s dolphins and harbour porpoises in the waters surrounding Bardsey Island had different distributions and habitat-use patterns. We first used kernel density grids to determine the core areas for both species, an approach previously used to define important areas for cetaceans using satellite telemetry data [17], [38], boat-based data [39] and land-based data [40]. The kernel grids showed that the two species use the local spatial area in different ways with Risso’s dolphins mainly using a core area to the West and porpoises mainly using a core area to the East of the Island (Figure 4). In addition, there is an area within the Sound where both species overlap in September. Secondly, we used GAMs to analyse the distribution of each species in relation to both dynamic cyclic and topographic variables, and variables most likely related to sighting conditions. The observation site and sea state were retained in the habitat models for both species, and these capture a large part of the heterogeneity in detection probability. Other variables can influence the detection probability (e.g. swell, water colour, cloud cover, and glare), but these were not included in the analysis. Although this may result in some unexplained variability, it probably does not heavily bias the results because variation of these variables within a sighting region was relative small, and the extensive observation effort (8261 scans, spread over 7 years), will further reduce its effect on the observed distribution of marine mammal sightings. Also group-size can influence the probability of detection (i.e. larger groups are often more easily detected). However this was not evident in the data when creating accumulation curves with a function for group-size (data not shown). Dynamic cyclic variables (seasonal, diel/diurnal, tidal and lunar cycles) were clearly identified as important features that influence the fine-scale distribution of these species. Each species showed different preferences and these are discussed below.

### Harbour Porpoise

For the harbour porpoise, sea state explained most variability in sighting rate. Although there may be a biological mechanism underlying this, it is most likely the consequence of sea state dependent detection probability. This effect seems relative substantial, with a 5 times lower probability of detecting porpoises during a sea state of 2 compared to 0. The fact that the porpoise presence was affected by sea state is consistent to other surveys [41]–[43]. The second most important variable was a smooth interaction between X and Y coordinates. Although a large number of spatial covariates were included in this study, this result implies that some process that drives the porpoise distribution is not included and that the other (physical) covariates are insufficient surrogates for this process. It is generally assumed that most relationships with such variables are indirect and are mediated through the habitat preferences of preferred prey species [2]. However, the direct relationship between predator distribution and its preferred prey may not necessarily be straight forward as might be anticipated for some species [44]. Nevertheless, some direct links were recently shown in the Baltic Sea between porpoise distribution and their prey [45], [46] but such data are difficult to collect at a fine-scale or temporal level.

The present study showed a relationship between topographic variables and porpoise distribution, involving depths with the highest sighting rate occurring in areas of approximately 14 m or >30 m depth, and those areas with North-facing and steep slopes. In UK waters, cetacean-habitat relationships have been explored for porpoises, and depth has been successfully used to explain distribution patterns [42], [43], [47], [48]. Seabed slope has also been found to influence porpoise distribution [18], [21], [49]. Porpoises in other studies showed a low preference for shallow (<20 m) waters [21], [49], [50]–[52] which is not consistent with our findings. The bottom topography in the area to the East of the Island demonstrates a small ‘gully’, with depths varying between 10 m and 20 m (Supplementary Figure S6). Such areas probably act as a restricted channel and interrupt the water flow and therefore may create areas where zooplankton accumulates and where fish may shelter from strong currents [53]. Similar observations were made off Angelsey where an area of the flood race demonstrated particularly high relief with gullies with depths varying between 10 m and 30 m, and where porpoise presence was higher during flood [54]. Such areas may also form a natural trap where fish possibly get caught between the different dominating currents and this may be intensified by irregular bottom topography. For example, at HW−4.5 hrs the direction of the currents through the Sound is still SE. Because the Island is obstructing the general flow, some of the water passing south of the island rotates northward before decreasing in speed when LWS is reached. Such parallel ‘streams of water’ flowing in opposite direction were visible during the observations and in particular to the East and North of the Island and intensified until LWS.

Recently, studies that were carried out at a finer spatial scale, showed that tidal variables, such as tidal state, tidal speed or tidal height, also have an important influence on both the distribution (40,43,49] and behaviour [17], [20], [54] of porpoises. However, the preferred tidal phase or speed appears to vary across areas [17], [20], [21], [40], [43], [49], [54]. For example, porpoises off West Scotland preferred areas with high current speeds and generally prefer high tide [43], those occurring off Land’s End (Cornwall) preferred strong ebbing tidal flows [40], off Skomer Island (South Wales) they preferred conditions when the tide started to ebb [21] and those in Ramsey Sound (South Wales) preferred the entire ebb tidal phase [20]. The porpoises presence in our study peaked at HW-3, which reflects the period just after LWS (during which the currents changed direction from SE to NW) at the onset of the flood cycle. These tidal currents rapidly build in strength and ultimately may become too strong for porpoises to maintain a favourable foraging position. The porpoises however also appeared to take benefit of these strong currents and were frequently observed ‘hitch-hiking the current’ (traveling with fast speed following the tidal flow through the Sound).

Porpoise presence off West Scotland was found to be highest during slack phases of the tidal cycle [49] and off Anglesey (North Wales) at HW-3 [54] which match our findings. The porpoises were probably moving between foraging areas during different tidal states on either side of the Sound. Land-based observations carried out from the Lleyn Peninsula showed that porpoises were foraging off the most westerly headland (M. de Boer, pers. Obs.) but this is too great a distance to observe from Bardsey Island. In South Wales, porpoises have also been shown to move from either side of a channel during different tidal states [20].

The majority of the porpoise calves were sighted to the East of the Island and mainly from point D (70% of all calves) whereas calves were less often encountered to the West (1% from points B and C-1; 15% from point C-2) and to the Southeast (14%). The waters to the East were more sheltered, areas of upwelling were visible and tidal races were not as pronounced compared to the West. From the ADCP data it is evident that this area has overall weaker currents (Figure 2). Similar findings were reported off Ramsey in South Wales where female porpoises with dependent calves also preferred areas characterised by weaker currents [20]. Females may avoid areas where tidal currents are strongest because of a risk of separation from calves that might experience difficulty swimming against the tidal stream [20]. Indeed, the speed at which porpoises surfaced was mainly fast within Bardsey Sound where faster currents persisted whilst to the East of the Island porpoises were surfacing mainly slow.

The porpoises were more frequently seen at 2–3 days following neap tide. As for tidal cycles, it seems that lunar phase preference also appears to vary across areas with higher densities of harbour porpoises predicted during spring tides off West Scotland [49] and off Vancouver Island (Canada) [55] but no preferences for either spring or neap tides were apparent using acoustic data off Angelsey [54].

The Irish Sea is generally mixed in winter, but in spring and summer a complex patchwork of mixed and stratified areas develops [56]. As in most areas of the Irish Sea the tides are sufficiently energetic to mix and create a vertically homogeneous water column [57]. Areas where stratification occurs are those where increased water depths and weak tidal streaming prevent the generation of sufficient turbulent energy to maintain vertical mixing against the surface buoyancy flux in summer [23]. The fronts which mark the boundaries between mixed and stratified waters in summer are zones of enhanced primary production and they influence the distribution of plankton and zooplankton [58], and may create preferred foraging sites for marine mammals [30]. A tidal frontal system exists in the shallow Cardigan Bay area in summer although this is influenced by wind mixing [23]. Within stratifying regions, a tidal stratification value of 2.75 m^−2^ s^3^ has been shown to represent the locations of fronts, separating permanently-mixed water from seasonally-stratified regions [57]. Values between 2.3 and 2.75 m^−2^ s^3^ indicate regions that can switch between being mixed and stratified, depending on the phase of biweekly tidal currents; values between 2.75 and 3.5 m^−2^ s^3^ are regions likely to see spring-neap impacts on sub-surface primary production within the thermocline and represent areas that always remain stratified in summer [57]. Although, the waters around Bardsey are expected to be unstratified due to the presence of strong tidal currents, it appears that in some areas the waters are stratified. Our findings indicated that porpoises were more frequently seen in areas with a stratification value of 3.3 m^−2^ s^3^ which is similar to the findings reported for porpoises in a shallow area in the North Sea (3.56 m^−2^ s^3^) [30]. Most notably, the porpoises showed a peak in sighting rate at 2–3 days following a neap tide. Coastal waters generally show a stronger stratification particularly during neap tides upon which the phytoplankton biomass at the surface rises (with the developing stratification) reaching its maximum about 2–3 days after neap tide [57]. It therefore appears that porpoises occur in those areas where stratification is maximised. As recently suggested by Scott et al. [30] marine top predators are more likely to forage in different locations, defined to some extent by the level of stratification. *Log10 (h/U^3^)* is an inverse measure of tidal mixing normalised by the water depth (which explains some of the extreme values caused by current speeds that were equal to zero).

The porpoises in the present study were more frequently seen in August (Figure 6c). Seasonal variation in harbour porpoise habitat preference and distribution within European waters are poorly understood. Peaks in sightings during the summer may be indicative of better survey conditions in those months, although significant variations in seasonal distributions have been observed in the southern North Sea, indicating that animals aggregate seasonally in ‘hot spots’ within their range [59]. Within the UK, August and September have been proposed as the months with peak numbers of porpoise encounters [51] which matches our findings. Seasonal migrations in this species have also been documented in other geographical areas such as the German Baltic Sea with increased use of coastal areas during the summer months [60], [61]. Considering that habitat preferences are strongly linked to prey availability some changes might be related to the seasonal variations in diet [62].

### Risso’s Dolphin

The Risso’s dolphins mostly preferred areas with relatively low spatial variation in current speed. The ADCP data revealed flow structures at slack water that were consistent with the formation of tidal eddies to the West of the Island during the flood cycle and to the East of the island during the ebb cycle [12], [63]. This eddy overlaps with the core area for Risso’s dolphins (Supplementary Figure S5). It was expected that the presence of eddies and frontal areas would result in a preference of dolphins for areas with a high spatial variation in current speed but the opposite was found. This may be because the spatial and temporal resolution of the sightings or ADCP current sampling was insufficient. The kernel density plots showed that dolphins favoured the Sound during ebb (data not shown). Large areas with upwelling (slick domes of water on the surface) were frequently observed there. A higher sighting rate for dolphins occurred in areas with a tidal stratification of ∼2.7 m^−2^ s^3^ which has been shown to represent the locations of tidal fronts, separating permanently-mixed water from seasonally-stratified regions [57]. At fine spatial scales, tidal frontal systems appear to enhance the primary productivity and it is recognised that these features may provide predictable concentrations of prey [14], [15].

The diet of Risso’s dolphins consists primarily of cephalopods [64]. The lesser octopus *Eledone cirrhosa* has been predominantly found in the stomachs of Risso’s stranded in Wales, Scotland and southern England [65], [66]. The lesser octopus has been recorded in waters depths of up to 700 m, but is most common in water depths between 50 and 300 m with peaks in occurrence between early summer to autumn (June – October), especially in inshore waters [67]. The region in the direct vicinity of Bardsey is relative shallow (0–50 m), and this would be at the upper range of the lesser octopus distribution. Risso’s indeed avoid the very shallow regions (<20 m, see Figure 6). It is interesting to note that the lesser octopus is a normal and regular predator of large crustaceans caught in commercial traps [67]. This might explain the multiple observations of Risso’s dolphins foraging in the vicinity of lobster pots set off the NW point off Bardsey. Sports fishermen fishing within the Risso’s core area whilst dolphins were present, also reported catching octopus (M. de Boer, pers. comm.). However, MacLeod et al. [44] did not find a relationship between Risso’s dolphin occurrence and a model-based estimate of the distribution of the lesser octopus, but the spatial resolution of the study may have been insufficient. Risso’s may exploit very small patches (<∼10 m in size) of suitable prey habitat which is beyond the resolution of most studies (including MacLeod et al. [44]).

This study shows that Risso’s were more often observed in the late afternoon. Currently, little is known about the Risso’s diel activity patterns and descriptions of their seasonal and inter-annual movement patterns in UK Waters. Cetacean studies off California indicated that Risso’s dolphins show variable behavioural states during the day and probably forage at night [68]. A significant diel pattern was also shown in the echolocation activity of Risso’s dolphins in the Southern California Bight [69] and Risso’s dolphins off the Azores were mainly resting in the morning and in the afternoon [70]. The Risso’s dolphins in the present study were often seen spread out over a wider area with single or pairs of animals conducting long dives, which is indicative of foraging.

Risso’s dolphin sightings indicate possible year-round residency off NW Scotland. However, sightings are more frequent in this region over the summer and autumn months [66] but it is likely that the available datasets are biased by much greater survey effort in summer. Off Southern California, the seasonal and inter-annual variability in Risso’s dolphin occurrence is high with a peak occurrence in autumn of most years [69]. Year-round residency and inshore or offshore movements in response to warm and cold waters has been reported for this species off California [71]. In the present study no Risso’s dolphin sightings were made in July but seasonality was not selected as an influential variable in the model. A possible explanation for this is the relative low coverage of the C-1 and B study areas during July (largely due to unfavourable sighting conditions; Table 1). Risso’s dolphins may have been present but were actually not observed. Incidental boat-based records do exist for Risso’s dolphins off Bardsey in the month of July but generally more sightings are recorded in August and September [72].

A recent review on the global distribution of Risso’s dolphins highlight a preference for the continental shelf and slope waters to oceanic depths throughout the species’ range [73]. The highest sighting rate in the present study occurred in areas of approximately ∼25 m depth but the dolphins were also observed in waters as shallow as 7 m. Similar observations with Risso’s occurring in shallow waters were reported off NW Scotland (<30 m) [74]. Risso’s dolphins are usually found in deeper waters (1000 m) [75], [76] and in less deep waters of the continental slope [77]. Risso’s dolphins off the Azores are more frequently sighted in waters of 600 m [78], whilst most dolphin sightings off Scotland occurred in <200 m depth [79]. In this study, the dolphins preferred areas with steep South-facing slopes. Other studies (Mediterranean and Azores) also confirm the preference for steep slopes [75]–[77].

### Conclusions

Knowledge about the habitat selection of cetaceans and the biological and physical variables that underpin this selection is important to interpret their distribution patterns. Such information is relevant for designing measures to reduce impacts of present and future anthropogenic activities including the creation and management of protected areas [80]. An importance aspect of habitat models involves the identification of important habitat variables, the prediction of a species’ distribution patterns and areas that show high levels of usage. This has been used for different cetacean species in areas which were surveyed at a larger scale [4], [7]. Preference for short-lived, yet predictable, oceanographic features may go unnoticed in large-scale surveys that visit a given area only briefly. It is therefore also essential to model their habitat selection based on more continuous data and if possible include multiple years/seasons in order to understand the fine-scale temporal patterns that drive the distribution of a species. The key drivers in the habitat selection, however, remain unclear for most cetaceans, as the fine-scale changes in their habitat use have not been examined. In some cases, line-transect surveys have been carried out over a smaller area and over a number of years or seasons and this already provides more information regarding the relations between cetaceans and tidal variables [9], [17], [43], [49], [81]. Recently, Isojunno et al. [21] explored the use of temporally intensive data derived from Platforms of Opportunity in order to achieve a better fine-scale precision to study porpoises. Only a few studies have used land-based data on cetaceans in order to investigate their habitat-use [1] and using GLMs [3] or GAMs [40]. The present study used a fine-scale repeated/continuous land-based survey design and GAMs to provide a temporal insight into the importance of dynamic cyclic patterns on the fine-scale spatial distribution of two different cetacean species.

Our findings show that porpoises and Risso’s dolphins appeared to be integrally linked to dynamic cyclic variables with both species using different core areas on a temporary but predictable basis. Other studies have also found that different cetacean species, e.g. minke whale and harbour porpoise, may use the same fine-scale ‘island wake’ feature, but with both species using different aspects of that feature [9], [17]. The measure of tidal stratification was shown to be important with porpoises occurring in areas when stratification is maximised and dolphins using a different habitat which was less stratified. The prime conditions for foraging in these tidal stratified systems appeared to be related to the flood cycle (LWS and the onset of the flood phase). The number of porpoises furthermore peaked following a few days after the neap tidal phase (first and third quarter moon). This temporal variability implies that porpoises move between the Bardsey Island region and other areas. Single large scale surveys may not capture such spatio-temporal patterns.

Our conclusion is that by using a fine-scale repeated survey design together with ADCP data, we identified patterns that drive the patchy distribution of porpoises and Risso’s dolphins in a shallow Island system. The links between harbour porpoise and Risso’s dolphin distribution and topographic and dynamic cyclic variables has not been previously documented. In particular involving the variety of variables included in the present model, and beyond the resolution of most studies. Such dynamic patterns may form the initial basis for identifying potentially critical habitats for these species within relatively shallow coastal systems. The information provided on how environmental characteristics determine a critical habitat serve as a blueprint for studies carrying out Environmental Impact Assessment studies related to planned anthropogenic activities in areas where cetaceans occur. Particularly, the expansion of marine renewable-energy developments, such as wind turbines, wave-power devices and tidal turbines, may negatively affect cetaceans in a variety of ways and often operate at a fine-spatial scale [82].

## Supporting Information

Figure S1 **Accumulation curves plotted using different sightings data.** Sightings data for Risso’s dolphins pooled for lower (black) *vs* higher points (grey) is shown at the top with parallel lines showing an indication of corresponding inflection points. The bottom plot shows the differences in curves between the two sectors surveyed from point C (C1 *vs* C2) for harbour porpoise (HP) or Risso’s dolphin (RD).
(TIF)

Figure S2 **Examples of accumulation curves plotted using sightings data collected during different sea states.** Sightings data for Risso’s dolphins for point C1 (top) and harbour porpoises for point D (bottom) for sea states 0–3.
(TIF)

Figure S3 **The level of error from rounding to the closest half reticle as measured with binoculars.** The radial distance of up to 2800 m (the inflection point for the C-1 study area) is shown.
(TIFF)

Figure S4 **Visualisation of the predicted relative sighting rate per unit area and time.** (a) Harbour porpoise (a) and (b) Risso’s dolphins. The model predictions are based on the best model fitted to all data (see also tables S2 and S3). The highest values range from red, yellow, green, cyan, blue, magenta (low).
(TIFF)

Figure S5 **Kernel density utilisation grid for Risso’s dolphin during flood and sighting positions (circles).** Shown in relation to a simulated tidal eddie during flood, indicate the direction and strength of the currents, darkest shade of grey shows the 50% kernel core-area. Information regarding currents and eddies were derived from Neil (2008).
(TIF)

Figure S6 **Depth profile to the East of Bardsey showing a small ‘dip’ or ‘gully’.**
(TIFF)

Figure S7 **Density plot of environmental covariates values for the observed harbour porpoise (red bars) and control/availability locations (black bars).**
(TIF)

Figure S8 **Density plot of environmental covariates values for the observed Risso’s Dolphin (red bars) and control/availability locations (black bars).**
(TIF)

Table S1 **Inflection points defined for different sea states (SS) for Risso’s dolphins (RD) & harbour porpoises (HP).**
(DOCX)

Table S2 **Model summary harbor porpoise habitat selection model.**
(DOCX)

Table S3 **Model summary Risso’s dolphin habitat selection model.**
(DOCX)

Text S1 **Single species approach.** Exploring the effect of distance on the number of Risso’s dolphin and harbour porpoise sightings by plotting accumulation curves which showed the proportion of total number of sightings within a given distance.
(DOCX)

Text S2 **Inter-species comparisons.** Exploring the difference in detection between Risso’s dolphin and harbour porpoise using accumulation curves.
(DOCX)
