# Supplementary figures and images for: The Influence of Topographic and Dynamic Cyclic Variables on the Distribution of Small Cetaceans in a Shallow Coastal System
Source: PLoS One. 2014 Jan 22;9(1):e86331. doi: 10.1371/journal.pone.0086331 (PMC3899228; doi:10.1371/journal.pone.0086331)

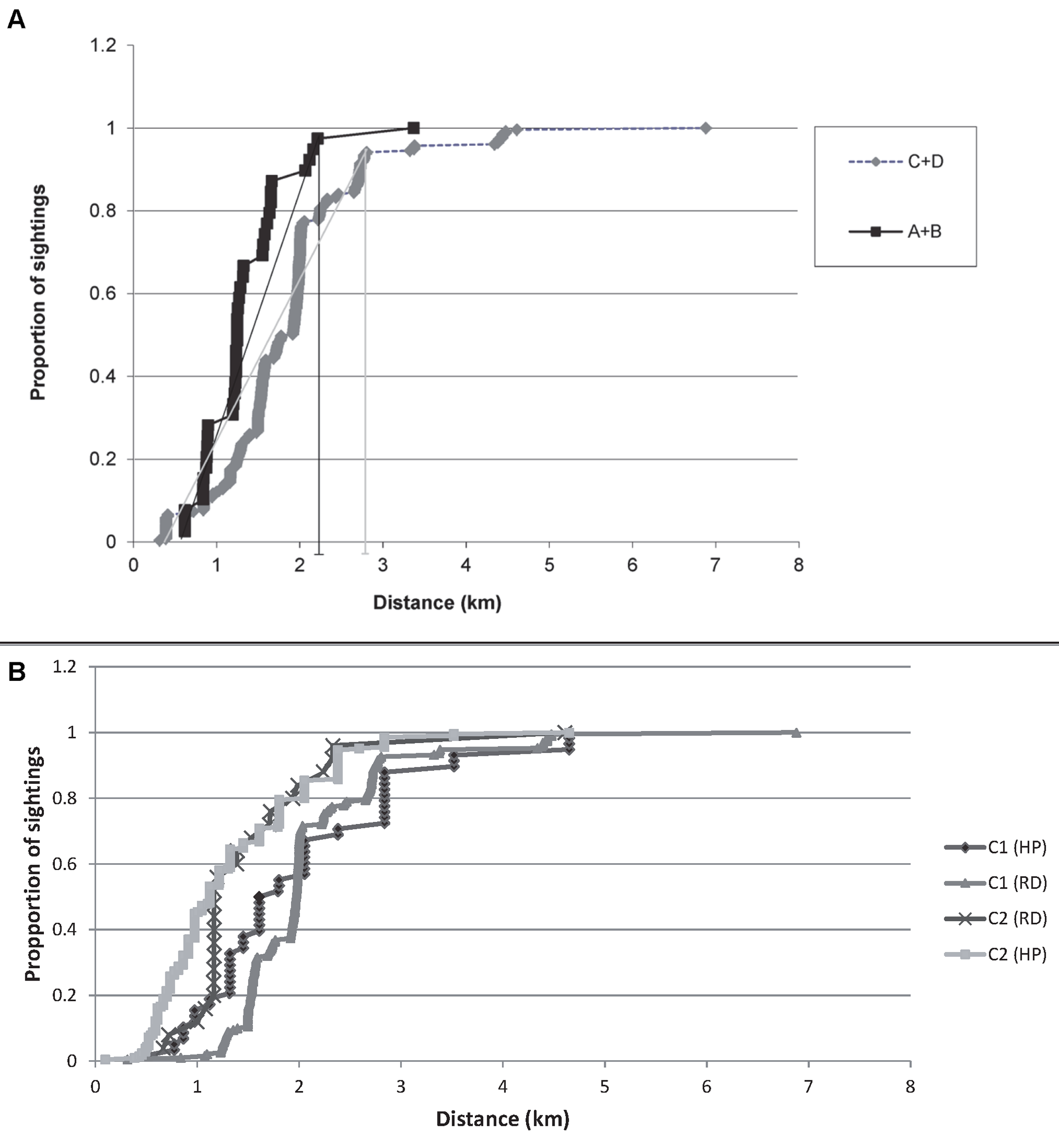

Supplement: Figure S1 — Accumulation curves plotted using different sightings data. Sightings data for Risso’s dolphins pooled for lower (black) vs higher points (grey) is shown at the top with parallel lines showing an indication of corresponding inflection points. The bottom plot shows the differences in curves between the two sectors surveyed from point C (C1 vs C2) for harbour porpoise (HP) or Risso’s dolphin (RD). (TIF) [file pone.0086331.s001.tif]

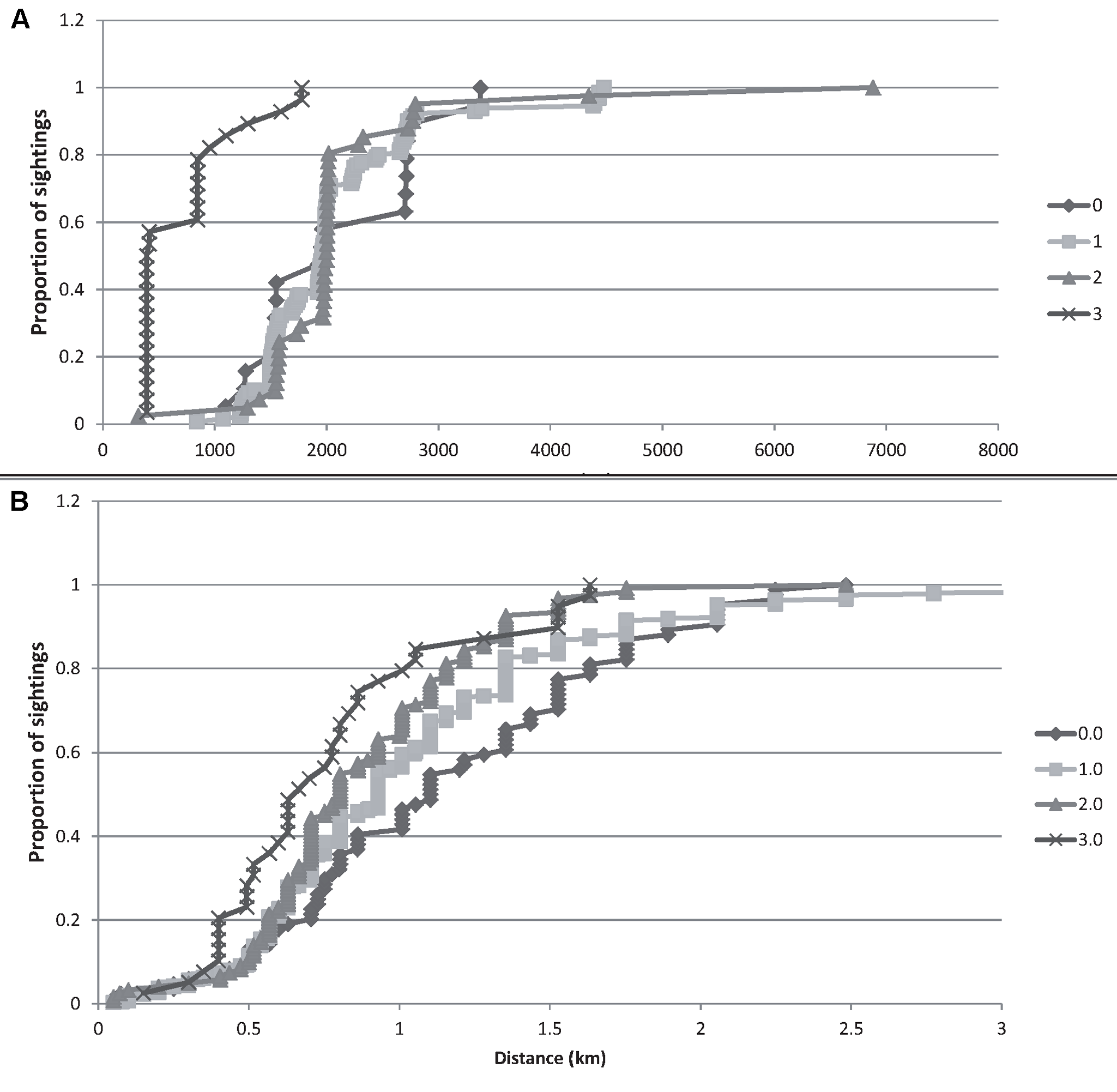

Supplement: Figure S2 — Examples of accumulation curves plotted using sightings data collected during different sea states. Sightings data for Risso’s dolphins for point C1 (top) and harbour porpoises for point D (bottom) for sea states 0–3. (TIF) [file pone.0086331.s002.tif]

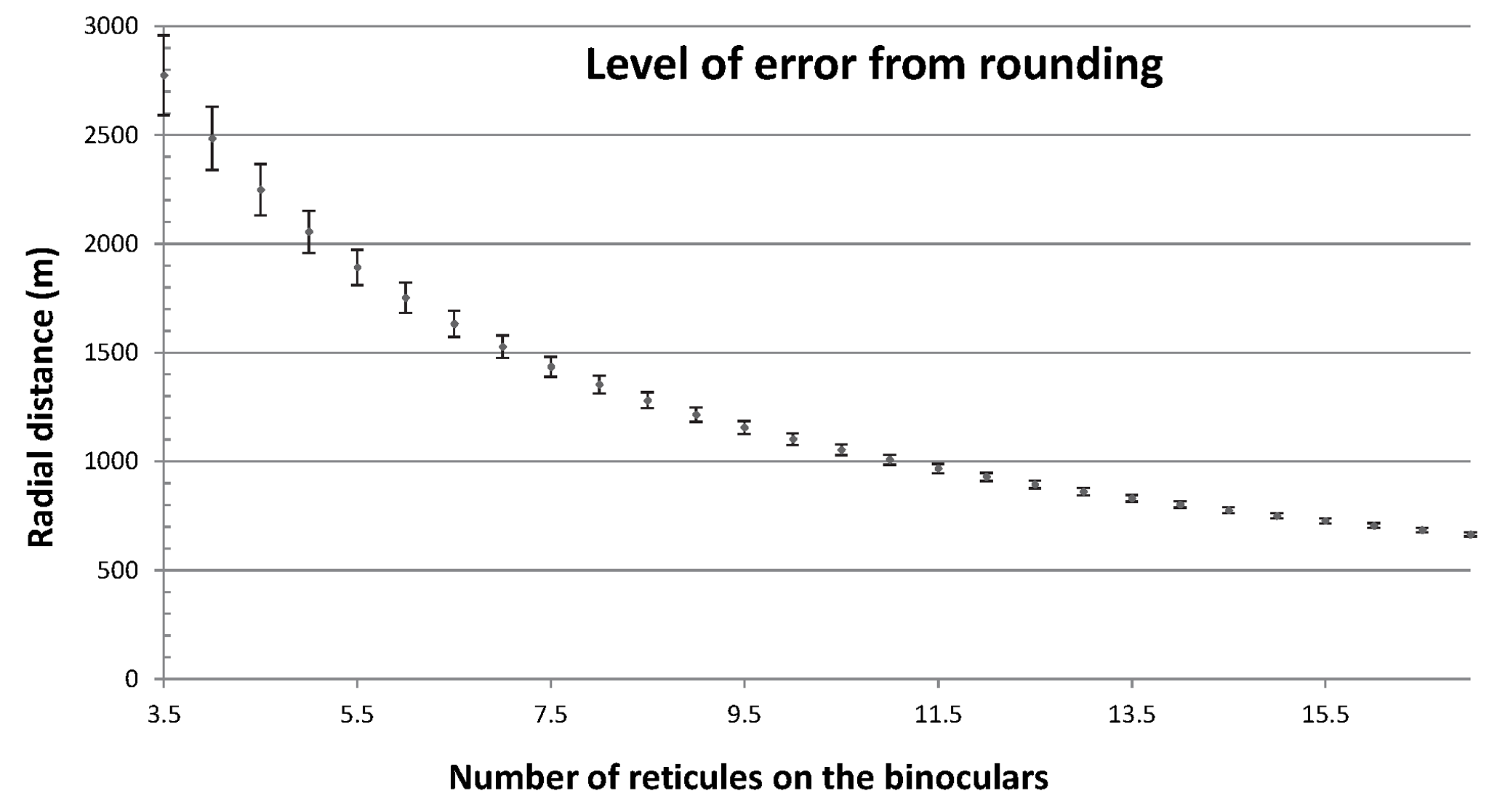

Supplement: Figure S3 — The level of error from rounding to the closest half reticle as measured with binoculars. The radial distance of up to 2800 m (the inflection point for the C-1 study area) is shown. (TIFF) [file pone.0086331.s003.tiff]

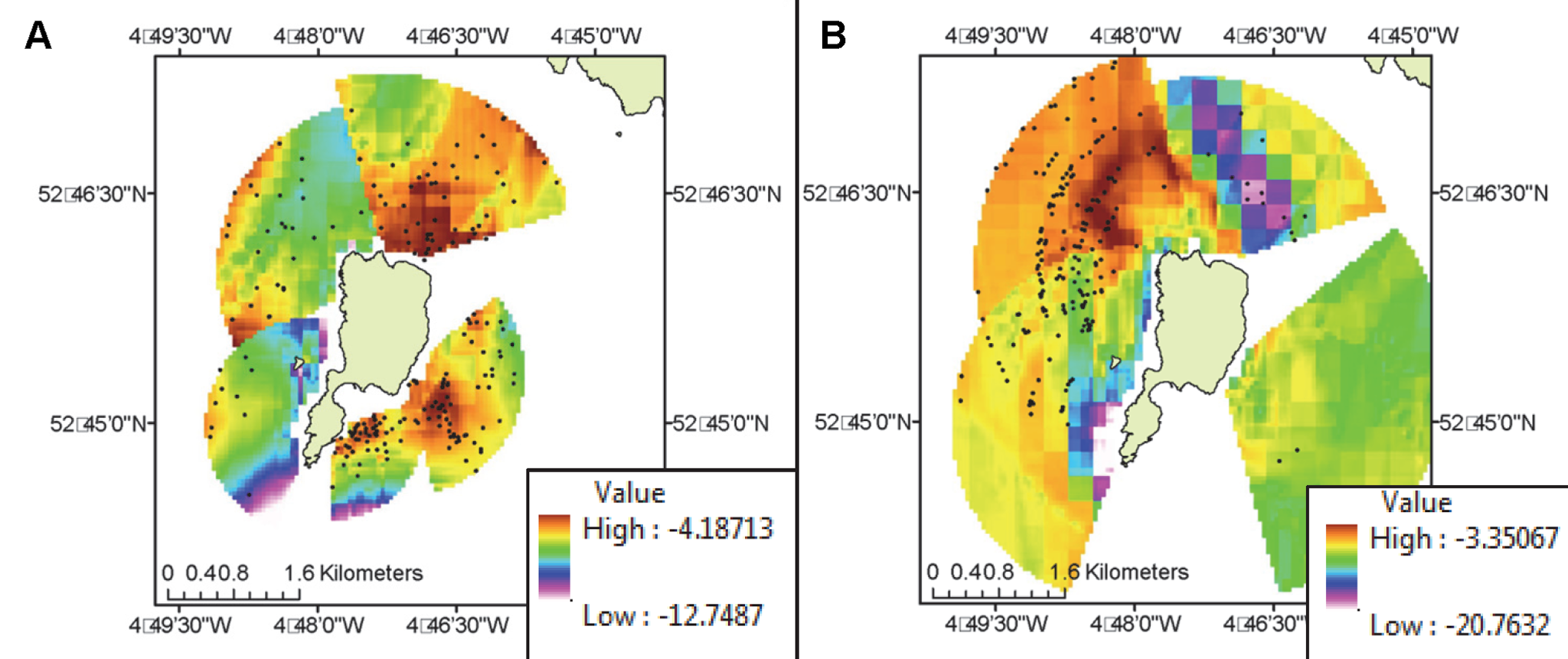

Supplement: Figure S4 — Visualisation of the predicted relative sighting rate per unit area and time. (a) Harbour porpoise (a) and (b) Risso’s dolphins. The model predictions are based on the best model fitted to all data (see also tables S2 and S3). The highest values range from red, yellow, green, cyan, blue, magenta (low). (TIFF) [file pone.0086331.s004.tiff]

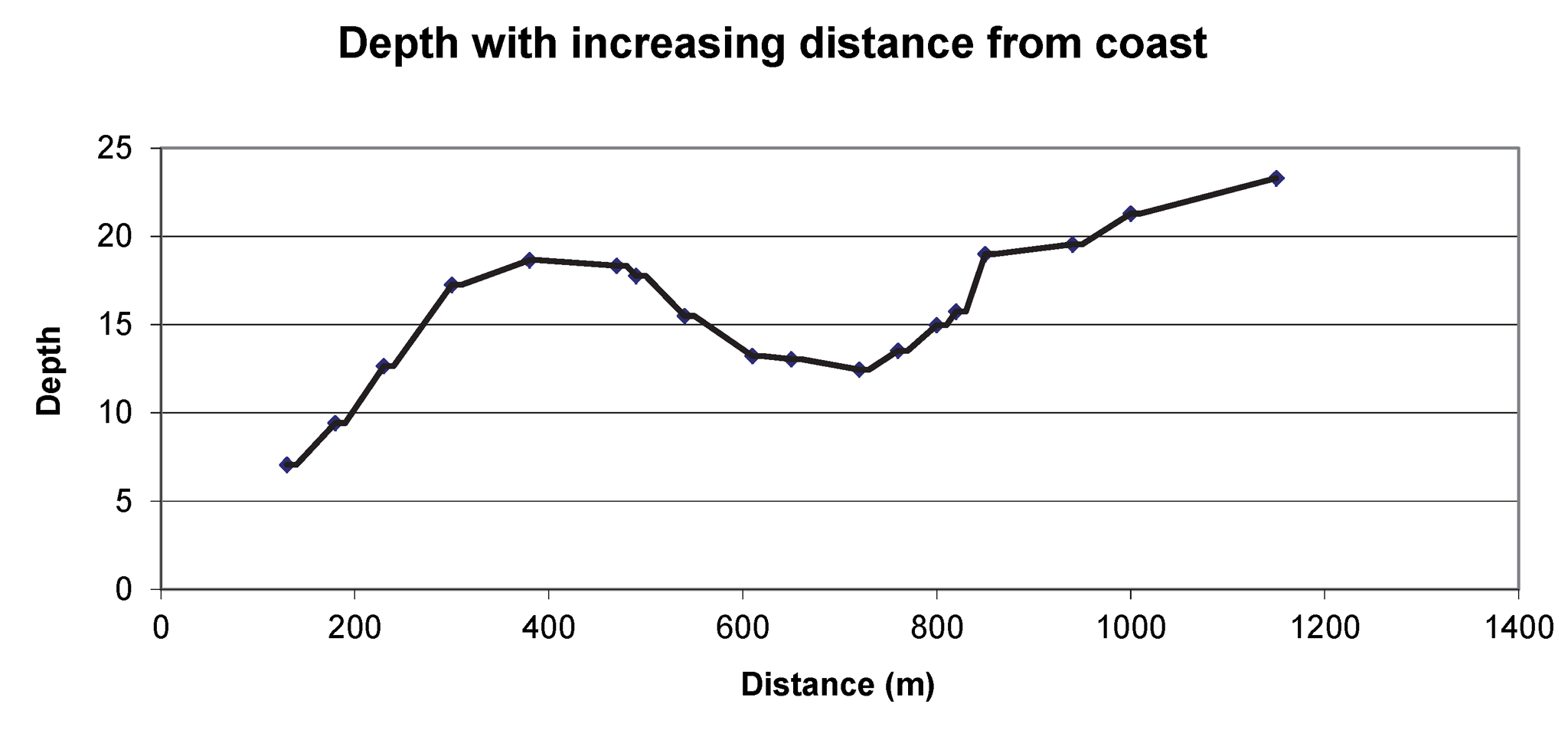

Supplement: Figure S6 — Depth profile to the East of Bardsey showing a small ‘dip’ or ‘gully’. (TIFF) [file pone.0086331.s006.tiff]

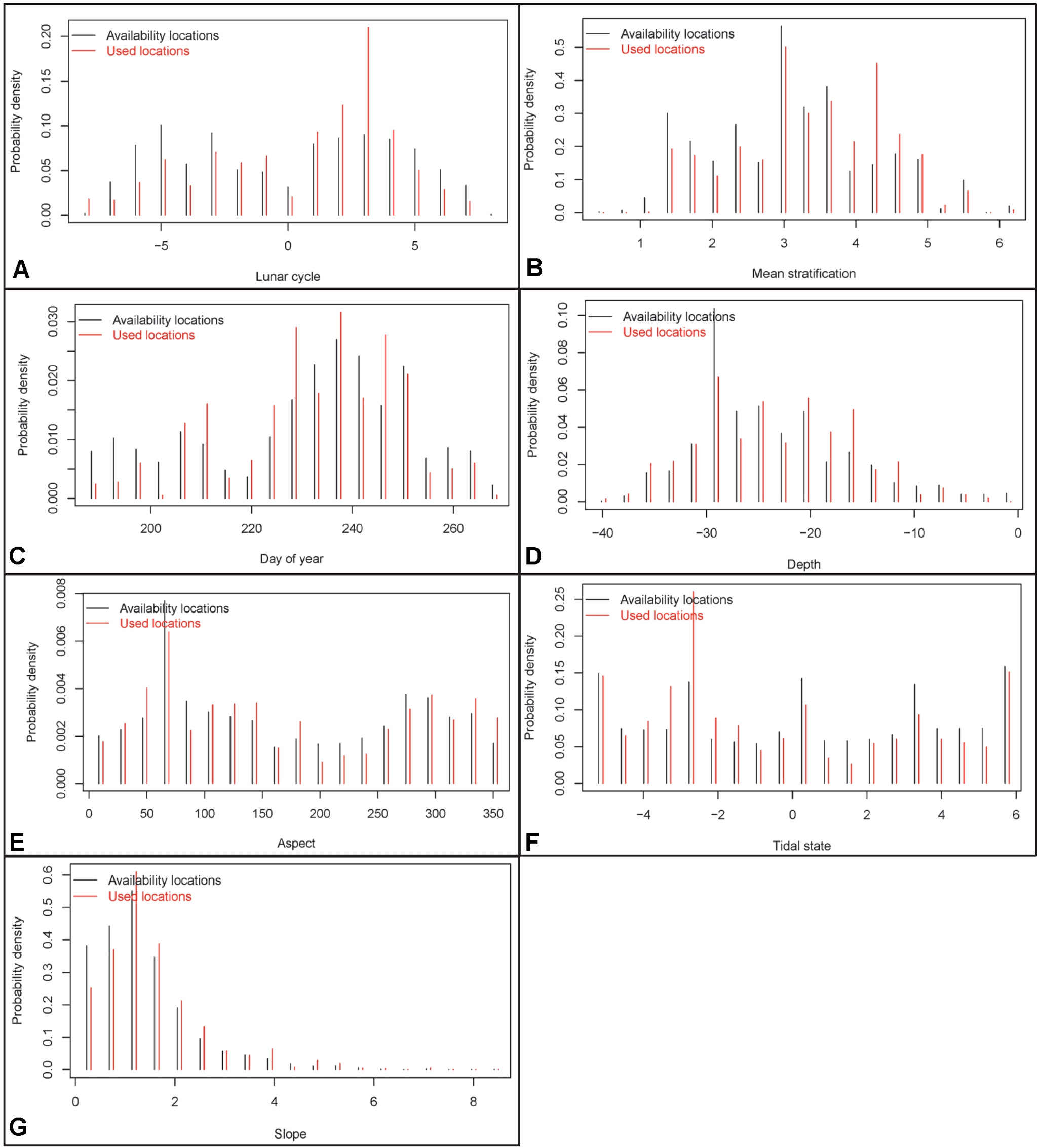

Supplement: Figure S7 — Density plot of environmental covariates values for the observed harbour porpoise (red bars) and control/availability locations (black bars). (TIF) [file pone.0086331.s007.tif]

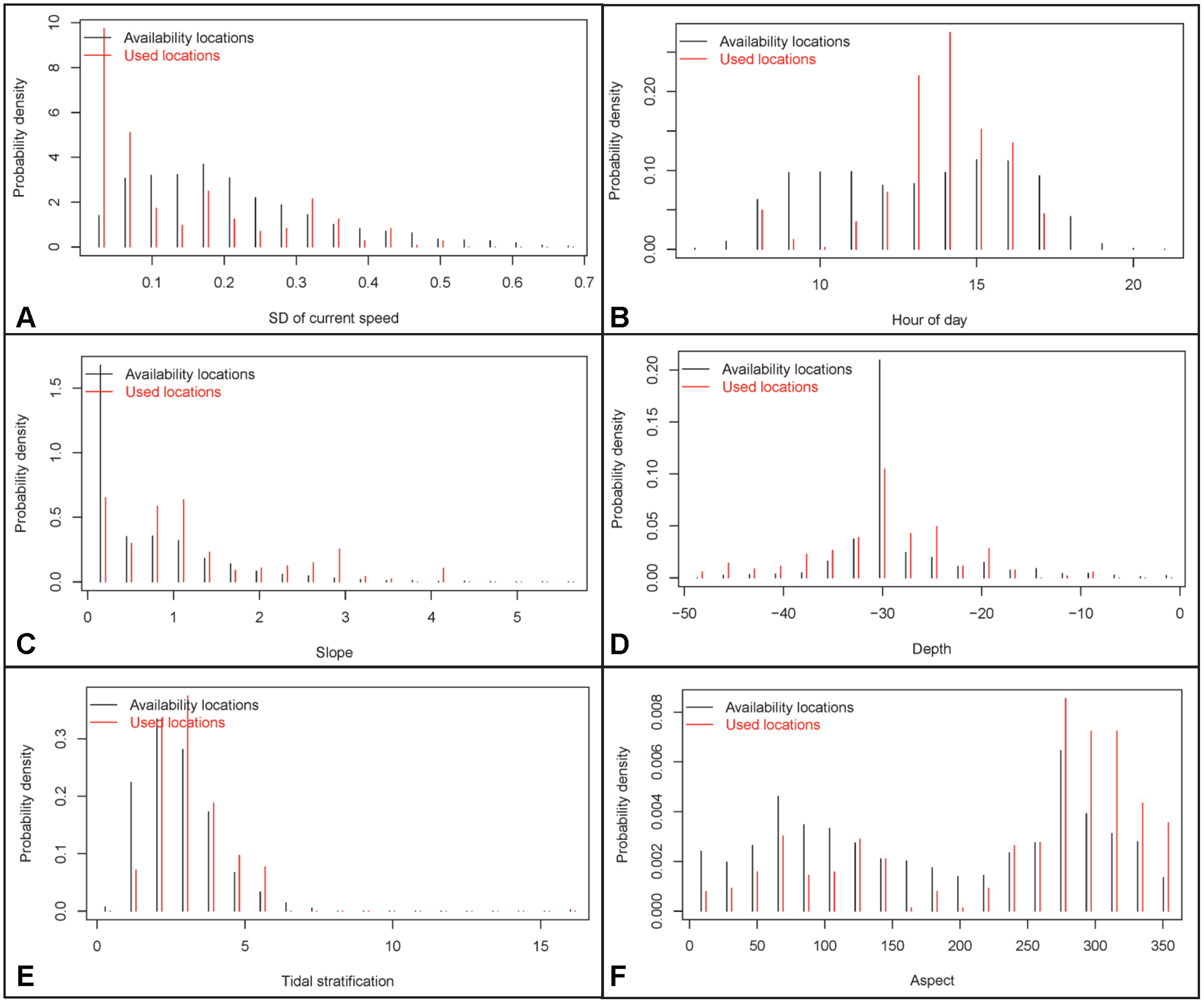

Supplement: Figure S8 — Density plot of environmental covariates values for the observed Risso’s Dolphin (red bars) and control/availability locations (black bars). (TIF) [file pone.0086331.s008.tif]
