# Supplementary material for: The Influence of Topographic and Dynamic Cyclic Variables on the Distribution of Small Cetaceans in a Shallow Coastal System
Source: PLoS One. 2014 Jan 22;9(1):e86331. doi: 10.1371/journal.pone.0086331 (PMC3899228; doi:10.1371/journal.pone.0086331)
Supplement: Table S1 — Inflection points defined for different sea states (SS) for Risso’s dolphins (RD) & harbour porpoises (HP). (DOCX) [file pone.0086331.s009.docx]

**Table S1** Inflection points defined for different sea states (SS) for Risso’s dolphins (RD) & harbour porpoises (HP).

| **Species** | **Survey-site** | **SS 0** | **SS 1** | **SS 2** | **SS 3** | **SS 0-2** |
| --- | --- | --- | --- | --- | --- | --- |
| RD | A | n/a | n/a | n/a | n/a | 2.167^a^ |
| HP | A | n/a | 1.092 | 1.053 | n/a | 1.053 |
| RD | B(B1+B2) | n/a | 2.216 | 2.167 | n/a | 2.167 |
| HP | B(B1+B2) | 2.253 | 1.552 | 1.318 | 0.982 | 1.318 |
| RD | C1 | 2.765 | 2.772 | 2.725 | 0.847 | 2.765 |
| HP | C1 | 2.052 | 2.052 | 2.052 | n/a | 2.052 |
| RD | C2 | n/a | 2.33 | 2.237 | n/a | 2.33 |
| HP | C2 | 2.382 | 2.382 | 1.323 | 0.815 | 2.382 (SS 0-1); 1.323 (SS 2) |
| RD | D | n/a | 2.054 | n/a | n/a | 2.765^b^ |
| HP | D | 1.817 | 1.478 | 1.329 | 1.053 | 1.478 (SS 0-1); 1.329 (SS 2) |

^a^ due to low sample size this is based on point B; ^b^ due to low sample size this is based on point C.
