# Supplementary material for: The Influence of Topographic and Dynamic Cyclic Variables on the Distribution of Small Cetaceans in a Shallow Coastal System
Source: PLoS One. 2014 Jan 22;9(1):e86331. doi: 10.1371/journal.pone.0086331 (PMC3899228; doi:10.1371/journal.pone.0086331)
Supplement: Table S2 — Model summary harbor porpoise habitat selection model. (DOCX) [file pone.0086331.s010.docx]

**Table S2.** Model summary harbor porpoise habitat selection model.

| **Parametric coefficients** | | | | |
| --- | --- | --- | --- | --- |
|  | Estimate | Std. Error | Z value | **Pr(>\|z\|)** |
| (Intercept) | -7.05852 | 0.15813 | -44.636 | < 2e-16 *** |
| factor(SEA)1 | -0.58310 | 0.07978 | -7.309 | 2.69e-13 *** |
| factor(SEA)2 | -1.68900 | 0.09211 | -18.337 | < 2e-16 *** |
| factor(SITE.NAME)A | -0.15776 | 0.21456 | -0.735 | 0.46216 |
| factor(SITE.NAME)B | -1.64830 | 0.60852 | -2.709 | 0.00675 ** |
| factor(SITE.NAME)C_1 | 0.99233 | 0.50384 | 1.970 | 0.04889 * |
| factor(SITE.NAME)C_2 | 3.10403 | 0.31498 | 9.855 | < 2e-16 *** |
| **Approximate significance of smooth terms** | | | | |
|  | **Edf** | **Ref.df** | **Chi.sq** | **p-value** |
| te(xuk,yuk) | 13.546 | 14.322 | 285.84 | < 2e-16 *** |
| s(LUNAR) | 1.958 | 1.999 | 137.13 | < 2e-16 *** |
| s(mean_stratification) | 2.964 | 2.999 | 67.93 | 1.18e-14 *** |
| s(yday) | 2.900 | 2.992 | 63.46 | 1.06e-13 *** |
| s(depth) | 2.919 | 2.994 | 50.72 | 5.57e-11 *** |
| s(aspect) | 1.966 | 1.998 | 53.72 | 2.16e-12 *** |
| s(HW.TIDAL) | 1.943 | 1.998 | 57.31 | 3.58e-13 *** |
| s(slope) | 2.706 | 2.940 | 19.25 | 0.000226 *** |

R-sq.(adj)= -0.353; Deviance explained = 7.51%; UBRE score = 0.20401;Scale est. = 1; n = 17305; ^*^Signif. codes: 0 ‘***’ 0.001 ‘**’ 0.01 ‘*’ 0.05 ‘.’ 0.1 ‘ ’ 1
