# Supplementary material for: The Influence of Topographic and Dynamic Cyclic Variables on the Distribution of Small Cetaceans in a Shallow Coastal System
Source: PLoS One. 2014 Jan 22;9(1):e86331. doi: 10.1371/journal.pone.0086331 (PMC3899228; doi:10.1371/journal.pone.0086331)
Supplement: Table S3 — Model summary Risso’s dolphin habitat selection model. (DOCX) [file pone.0086331.s011.docx]

**Table S3.** Model summary Risso’s dolphin habitat selection model.

| **Parametric coefficients** | | | | |
| --- | --- | --- | --- | --- |
|  | **Estimate** | **Std. Error** | **Z value** | **Pr(>\|z\|)** |
| (Intercept) | -11.1936 | 0.3045 | -36.766 | < 2e-16 *** |
| factor(SITE.NAME)B | 2.2585 | 0.2471 | 9.141 | < 2e-16 *** |
| factor(SITE.NAME)C_1 | 3.6032 | 0.2207 | 16.327 | < 2e-16 *** |
| factor(SITE.NAME)C_2 | 2.1870 | 0.2257 | 9.691 | < 2e-16 *** |
| factor(SEA)1 | 0.6553 | 0.2421 | 2.707 | 0.00679 ** |
| factor(SEA)2 | -0.6697 | 0.2588 | -2.588 | 0.00966 ** |
| **Approximate significance of smooth terms** | | | | |
|  | **edf** | **Ref.df** | **Chi.sq** | **p-value** |
| s(sd_speed) | 2.973 | 3.000 | 70.58 | 3.20e-15 *** |
| s(hour) | 2.973 | 2.999 | 117.61 | < 2e-16 *** |
| s(slope) | 1.000 | 1.001 | 76.71 | < 2e-16 *** |
| s(depth) | 2.935 | 2.996 | 34.17 | 1.82e-07 *** |
| s(tidal_stratification) | 2.891 | 2.993 | 38.69 | 2.00e-08 *** |
| s(aspect) | 1.911 | 1.996 | 28.83 | 5.45e-07 *** |

R-sq.(adj)= -0.456; Deviance explained = 19.7%; UBRE score = -0.6;Scale est. = 1; n = 14876; ^*^Signif. codes: 0 ‘***’ 0.001 ‘**’ 0.01 ‘*’ 0.05 ‘.’ 0.1 ‘ ’ 1
