# Supplementary material for: The Influence of Topographic and Dynamic Cyclic Variables on the Distribution of Small Cetaceans in a Shallow Coastal System
Source: PLoS One. 2014 Jan 22;9(1):e86331. doi: 10.1371/journal.pone.0086331 (PMC3899228; doi:10.1371/journal.pone.0086331)
Supplement: Text S1 — Single species approach. Exploring the effect of distance on the number of Risso’s dolphin and harbour porpoise sightings by plotting accumulation curves which showed the proportion of total number of sightings within a given distance. (DOCX) [file pone.0086331.s012.docx]

**Text S1 Single species approach**

We studied the effect of distance on the number of Risso’s dolphin sightings by plotting the accumulation curve which showed the proportion of total number of sightings within a given distance for the lower observation points (A+B) and compared these to the higher points (C+D). As expected, the inflection point for the higher platforms differed (2.8km) to that for the lower ones (2.2 km; Fig. S1). It was also found that the accumulation curves for Risso’s differed for point C (C1 *vs* C2) and it was decided to treat these two survey sectors separate because of their different inflection points (Fig. S1). The accumulation curves for both sectors (B1 and B2) covered from point B were comparable and we concluded that data could be pooled (figures not shown). Similar results were found for porpoises (Fig. S1).

We then explored how the sea state was affecting the accumulation curve for both species. From figure 3 it is evident that for dolphins the sea states 0-2 followed a similar accumulation curve but that this differed for sea state 3 (Fig. S2). We therefore only include sea states 0-2 for any further data analysis regarding dolphins. One might argue that the accumulation curve for sea state 2 for porpoises does not quite follow a similar curve compared to lower sea states however for sea state 3 this is more pronounced (Fig. S2). For the higher points (C+D), we concluded to pool all porpoise sightings made during sea states 0-1 up to the defined inflection point but only to include those observations made during sea state 2 up to the corresponding inflection point. The distance (based on the defined inflection points) to which we assume that the number of sightings remained constant are listed in Table S1 for each of the different survey sectors and for both species.
