# Supplementary material for: The Influence of Topographic and Dynamic Cyclic Variables on the Distribution of Small Cetaceans in a Shallow Coastal System
Source: PLoS One. 2014 Jan 22;9(1):e86331. doi: 10.1371/journal.pone.0086331 (PMC3899228; doi:10.1371/journal.pone.0086331)
Supplement: Text S2 — Inter-species comparisons. Exploring the difference in detection between Risso’s dolphin and harbour porpoise using accumulation curves. (DOCX) [file pone.0086331.s013.docx]

**Text S2 Inter-species comparisons**

For point C1 and C2 we noticed a different accumulation curve (Fig. S1) and this indicated that the two sectors potentially differ in physical habitat. It seems likely that the relatively narrow and deep channel (Bardsey Sound) is responsible for this difference as the inflection point for both Risso’s dolphins and porpoises is noticeably shorter for C2. There is also a difference in detection between both species for point D, with a much higher inflection point measured for Risso’s dolphins compared to porpoises, however, however, this is based on a low sample size of dolphins (data not shown).
